# Checkpoint Blockade Rescues the Repressive Effect of Histone Deacetylases Inhibitors on γδ T Cell Function

**DOI:** 10.3389/fimmu.2018.01615

**Published:** 2018-07-19

**Authors:** Sajad A. Bhat, Disha Mohan Vedpathak, Shubhada V. Chiplunkar

**Affiliations:** ^1^Chiplunkar Laboratory, Advanced Centre for Treatment, Research and Education in Cancer (ACTREC), Tata Memorial Centre, Navi Mumbai, India; ^2^HomiBhabha National Institute, Mumbai, India

**Keywords:** gamma delta (γδ) T cells, phosphoantigen, histone deacetylases inhibitors, effector functions, programmed death-1, programmed death ligand-1

## Abstract

Histone deacetylases (HDAC) are one of the key epigenetic modifiers that control chromatin accessibility and gene expression. Their role in tumorigenesis is well established and HDAC inhibitors have emerged as an effective treatment modality. HDAC inhibitors have been investigated for their specific antitumor activities and also clinically evaluated in treatment of various malignancies. In the present study, we have investigated the effect of HDAC inhibitors on the effector functions of human γδ T cells. HDAC inhibitors inhibit the antigen-specific proliferative response of γδ T cells and cell cycle progression. In antigen-activated γδ T cells, the expression of transcription factors (Eomes and Tbet) and effector molecules (perforin and granzyme B) were decreased upon treatment with HDAC inhibitors. Treatment with HDAC inhibitors attenuated the antitumor cytotoxic potential of γδ T cells, which correlated with the enhanced expression of immune checkpoints programmed death-1 (PD-1) and programmed death ligand-1 in γδ T cells. Interestingly, PD-1 blockade improves the antitumor effector functions of HDAC inhibitor-treated γδ T cells, which is reflected in the increased expression of Granzyme B and Lamp-1. This study provides a rationale for designing HDAC inhibitor and immune check point blockade as a combinatorial treatment modality for cancer.

## Introduction

Gamma delta T cells, the enigmatic brethren of alpha beta (αβ) T cells were discovered coincidently during cloning the αβ T-cell receptor (TCR) locus (1). This small subset of T cells, γδ T cells constitute about 5–10% of the circulating T cell population, which express the variant form of TCR heterodimer (2). γδ T cells manifest the features of both innate and adaptive immunity (3). TheVγ9Vδ2 T cell subset of γδ T cells predominates in peripheral blood, and these cells play an important role in the defense against microbial pathogens, stressed cells, and tumor cells of various origin (4, 5). γδ T cells differ from αβ T cells by their TCR gene usage, tissue tropism, and MHC-independent antigen recognition (6, 7). γδ T-cells display broad functional plasticity, like regulatory potential, antigen-presenting capacity, B-cell helper activity, and have the potential for diverse cytokine production (8). γδ T cells recognize non-peptide phosphoantigens such as isopentenyl pyrophosphate (IPP) or 4-hydroxy-3-methyl-but-2-eneyl pyrophosphate (HMBPP), which are produced through the mevalonate pathway in mammalian cells or non-mevalonate/Rohmer pathway in non-mammalian cells, respectively (9). γδ T cells are also activated indirectly by aminobisphosphonates such as Zoledronate. Aminobisphosphonates inhibit the key enzyme of mevalonate pathway, farnesyl pyrophosphate synthase and lead to accumulation of IPP. Tumor cells treated with aminobisphophonates show increase in the intracellular level of IPP and, therefore, are easily targeted by γδ Tcells (10, 11).

Activated γδ T cells are known to produce large amounts of the pro-inflammatory cytokines interferon-γ (IFN-γ) and tumor necrosis factor-α (TNF-α) as well as the chemokines MIP-1 (macrophage inflammatory protein) and RANTES (regulated on activation, normal T cell expressed, and secreted) (12). In addition, cytolytic mediators such as granzyme B and perforin are produced to induce specific lysis of cells with elevated phosphoantigen levels (13). Transcription factors like Eomes and Tbet are known to be expressed upon activation by γδ T cells and are essential for antitumor effector functions (14).

Nucleosome is the basic structure of eukaryotic chromatin, composed of histones and DNA. Each nucleosome comprises 146 bp of DNA wrapped around an octamer of core histones (two H2A–H2B dimers and a H3–H4 tetramer) (15). Histone proteins are rich in basic amino acids lysine and arginine. It is through interaction with these histone proteins that massive DNA is packed inside the nucleus. The tails of histone proteins undergo different complex and coordinated posttranslational modifications like histone acetylation, methylation, phosphorylation, and ubiquitination. According to histone code hypothesis, these modifications are read by specific factors, which ultimately lead to downstream events (16). Histone modifications are reversible in nature and influence many fundamental biological processes. Histone acetylation are directed by histone modifying enzymes, histone acetyl transferases (HAT), and histone deacetylases (HDAC), which participate in potential cross-talk between different modifications (15). Normal physiological functions require a balance between HAT and HDAC. Abrupt alterations that skew this balance can give rise to different pathophysiological conditions like cancer (17, 18).

Histone deacetylases inhibitors, including Trichostatin-A (TSA) and sodium valproate (VPA), can alter the acetylation of histones in chromatin and enhance gene transcription. In the recent decades HDAC inhibitors have received attention as anti-neoplastic treatment. Extensive evidence suggests that HDAC inhibitors play a role in antitumor immunity (19). HDAC inhibitors lead to growth arrest, induction of apoptosis, and differentiation in tumors. Pan HDAC inhibitors like VPA, TSA, and suberoylanilidehydroxamic acid (SAHA) target Class I (HDAC 1, 2, 3, and 8), Class II (HDAC 4, 5, 7, 9, 6, and 10) HDACs. Hence, their anticancer activities are pleotropic in nature, mediated by altering the expression of various genes that are regulated by class I and II HDACs. Additionally, they also target several non-histone proteins such as transcription factors (p53, E2f1) and cytoplasmic proteins (tubulin, hsp, β-catenin). Hyperacetylation of these histone and non-histone proteins brought about by HDAC inhibiton culminate in induction of cell-death pathways in cancer cells. Several studies have established effective tumor reduction *in vitro* as well as *in vivo* upon HDAC inhibitor treatment (20).

Moreover, HDAC inhibitors inhibit angiogenesis and increase the tumor cell antigenicity (21, 22). HDAC inhibitors mediate elevated expression of antigens on tumor cells so that they can be easily targeted by immune cells (23, 24). Due to these promising antitumor functions, HDAC inhibitors are now assessed in clinical trials and some of them have been approved for treatment (25, 26). Recent reports have demonstrated that HDAC inhibitors enhance response to immune checkpoint blockade in triple negative breast cancer, lung adenocarcinoma, melanoma, and multiple myeloma (27–30).

Although the impact of HDAC inhibitors on tumor cells is well studied, their effect on immune cells has recently surfaced. HDAC inhibitors have been shown to have a dual effect on immune cells, either enhancing their activation in cases of CD4 T cell and Tregs whereas dampening the effector functions of NK cells and CD8 T cells. HDAC inhibitors are also known to inhibit the cytotoxic potential of NK cells. HDAC inhibitors are also reported to downregulate the co-stimulatory molecules and cytokine signals in antigen-presenting cells (31). Previous studies have shown that HDAC inhibitor treated tumor cells are easily targeted by γδ T cells (32), but the impact of HDAC inhibitors on the functional responses of human γδ T cells are not well understood.

For successful immunotherapy, T cell responses are essential. Besides the TCR signal, co-stimulatory signal also determines the functional response of T cells. Co-stimulatory signal may be of positive or negative. Negative co stimulatory signals may be from different receptors like programmed death-1 (PD-1) and PD ligand-1 (PD-L1) interaction. PD-1 and PD-L1 are the members of immunoglobin family like that of CD28. Interaction of PD-1 and PD-L1 leads to functional impairment in T cells (33). It is well-known fact that tumors use this mechanism to escape the immune attack. Blocking antibodies for these immune check points can enhance antitumor responses, and these immune-modulating antibodies have achieved clinical success with FDA approved treatments for several malignancies (34). It has been shown that γδ T cells express PD-1 and PD-L1 and blocking of this signaling lead to increase in the antitumor potential of γδ T cells (35).

The present study focuses on investigating the direct impact of HDAC inhibitors on human γδ T cells. We have studied the effect of three different HDAC inhibitors, TSA, SAHA, and VPA on γδ T cells. We observed that HDAC inhibitors suppress the antigen-specific proliferative responses of γδ T cells and their antitumor effector functions by increasing the expression of immune checkpoints (PD-1 and PD-L1). The study further demonstrates that blocking of immune checkpoints on γδ T cells is capable of augmenting their antitumor cytotoxic potential. The present study will open new avenues in the field of cancer immunotherapy using HDAC inhibitors.

## Materials and Methods

### γδ T Cell Separation

Heparinized peripheral blood was collected from healthy individuals. Peripheral blood mononuclear cells (PBMCs) were isolated by differential density gradient centrifugation using Ficoll Hypaque (Sigma-Aldrich, St. Louis, MO, USA). The study was approved by the Institutional Ethics Committee (TMC-IECIII Project no. 166) and written informed consent was obtained from the donors prior to collection of blood samples. The experimental conditions and procedures for handling blood samples were performed as per the biosafety guidelines of the Institute Biosafety Committee. In short, blood samples were handled in biosafety cabinets and personnel handling blood samples were vaccinated against Hepatitis B. γδ T cells were purified from PBMCs using immunomagnetic MicroBeads (Miltenyi Biotech, Bergish Gladbach, Germany) by positive selection, as per manufacturer’s instructions. The purity of separated γδ T cells was >95% as confirmed by flow cytometry (FACS Aria, BD Biosciences, USA). Isolated γδ T cells were cultured in RPMI 1640 supplemented with 10% heat inactivated AB serum, 2 mM glutamine, and antibiotics.

### Cell Viability Assay

The viability of γδ T cells upon treatment with HDAC inhibitors was evaluated with MTT assay and Annexin V and 7-AAD staining. Briefly, 0.1 × 10^6^ γδ T cells, seeded in 96-well flat bottom plates (Nunc), were treated with the following HDAC inhibitors for the given concentration range: VPA (4–0.25 mM; Sigma-Aldrich), TSA (200–25 nM; Sigma-Aldrich), and SAHA (4–0.25 µM; Sigma-Aldrich) along with HDMAPP (1 nM; Echelon) and rIL-2 (50 IU/ml; Peprotech) for 72 h. γδ T cells treated only with HDMAPP (1 nM) and rIL-2 (50 IU/ml) were used as control. DMSO was used as vehicle control. Following 72 h of treatment, MTT (5 mg/ml) was added and incubated for 4 h at 37°C. After incubation, the spent medium was discarded, the formazan crystals were dissolved in DMSO, and the absorbance was measured at 570 nm by microplate reader (TECAN, Switzerland). Untreated γδ T cells were used as reference for calculating the viability. Concentrations of HDAC inhibitors, which had no impact on viability of γδ T cells were further validated by Annexin V and 7-AAD staining. The concentration of HDAC inhibitors showing viability more or equal to 90% in γδ T cells were used for all the further experimental procedures.

### Quantitative Real-Time PCR (qPCR)

The purified γδ T cells, activated with HDMAPP (1 nM) and rIL-2 (50 IU/ml) were treated in the presence or absence of HDAC inhibitors at the given concentrations VPA (2, 1, 0.5 mM), TSA (100, 50, 25 nM), and SAHA (1, 0.5, 0.25 µM) for 72 h. DMSO was used as vehicle control. Total cellular RNA was isolated by using Trizol reagent (Invitrogen Life Technologies, NY, USA) in accordance with the company’s instructions and cDNA was synthesized by High-Capacity cDNA Reverse transcription kit (Applied Biosystems) according to manufacturer’s instructions. The gene expression of T-bet, Eomes, perforin, granzyme B, IFN-γ, and TNF-α was evaluated by Quantstudio 15k Flex system (Applied Biosystems) using Power SYBR Green reagents (Applied Biosystems) as per manufacture’s procedure. All samples were analyzed with the following sequence specific primers: Perforin forward and reverse primer 5′-GACACACAAAGGTTCCTGCG-3′and5′-GACTTTGGCCCTGGTTACAT-3′, respectively, Granzyme B forward and reverse primer 5′-CAACCAATCCTGCTTCTGCT-3′ and 5′-GTCGTCTCGTATCAGGAAGC-3′, respectively, Eomes forward and reverse primer 5′-ATTCCACCGCCACCAAACTG-3′ and 5′-GCACCACCTCTACGAACAC-3′, respectively, Tbet forward and reverse primer 5′-GTGACCCAGATGATTGTGCT-3′ and 5′-ATGCGTGTTGGAAGCGTTGC-3′, respectively, IFN-γ forward and reverse primer5′-GCATCGTTTTGGGTTCTCTTG-3′ and 5′-AGTTCCATTATCCGCTACATCTG-3′, respectively, TNF-α forward and reverse primer 5′-ACTTTGGAGTGATCGGCC-3′ and 5′-GCTTGAGGGTTTGCTACAAC-3′, respectively, and 18S rRNA forward and reverse primer 5′-AACGGCTACCACATCCAA-3′ and 5′-TTCCAATTACACGGCCTC-3′, respectively. The gene expression was determined by threshold cycle (C_T_) method by applying 2^−ΔΔCt^. All the values were normalized to the expression of 18S rRNA as endogenous control.

### Western Blot Analysis

1 × 10^6^ freshly isolated γδ T cells were cultured with HDMAPP (1 nM), rIL-2 (50 IU/ml), and with or without HDAC inhibitors at the given concentrations VPA (2, 1, 0.5 mM), TSA (100, 50, 25 nM), and SAHA (1, 0.5, 0.25 µM) at 37°C. After 72 h of treatment, cells were harvested and whole cell lysates were prepared with SDS lysis buffer (1 M Tris–HCl pH 6.8, 10%w/v SDS, glycerol, β-mercaptoethanol, 1M DTT, and bromophenol blue). 10% Polyacrylamide gels were used to resolve the protein samples and transferred to Hybond-ECL nitrocellulose membrane (Amersham Pharmacia Biotech, Piscataway, NJ, USA). The primary antibodies to T-bet (1:1,000) (Cell Signalling Technology), p21 (1:1,000) (Abcam), Eomes (1:1,000) (Abcam), p53 (1:500) (Santa Cruz), NF-κB (1:1,000) (Abcam), total H3 (Abcam) (1:1,000), total H4 (Abcam) (1:1,000), acetyl H3 (Abcam) (1:1,000), acetyl H4 (Abcam) (1:1,000), and β-actin (1:4,000) (Sigma-Aldrich) as loading control were added at different dilution. Immunostaining was performed using appropriate secondary antibody at a dilution of 1:10,000 and developed with ECL plus Western blot detection system (Amersham Pharmacia).

### Immunostaining and Cell Cycle Analysis

The magnetically sorted γδ T cells were kept overnight in RPMI supplemented with 10% FBS and were stained for various cell surface markers such as Vδ2 TCR, CD14, CD19, and CD56. Briefly, the cells were harvested from culture, washed with ice cold PBS, and fixed with 1% paraformaldehyde at 4°C for 15 min. The cells were washed with FACS buffer and then labeled with fluorophore tagged antibodies Vδ2-PE, CD3-PECy7, CD14-PerCP, CD19-FITC, and CD56 PerCP Cy5.5 (BD Biosciences, USA) for 30 min at 4°C. Further, the cells were washed and acquired on FACS Aria (BD Biosciences, San Jose, CA, USA). γδT cells treated with or without HDAC inhibitors for 72 h as described earlier were stained with live–dead (LD) fixable dead cell stain kit (Thermo Fischer) as per manufacturer’s protocol. After staining with LD dye, the cells were fixed with paraformaldehyde and permeabilized with 1% saponin. Cells were washed and stained with γδ TCR-PE, CD25-PerCPCy5.5, CD69-APC (BD Biosciences, USA), Perforin-BV421, Granzyme B-PECF594, PD-1-PECF594, and PD-L1-PerCP Cy5.5 (BioLegend, San Diego, CA, USA), NKG2D-APC, CD16-BV421, KIRD2L2/3-PE (Miltenyi Biotech, Bergish Gladbach, Germany). For determination of degranulation marker, Lamp-1 (CD107a) and effector molecule Granzyme B release, purified γδ T cells were activated with rIL2 (50 IU/ml) and HDMAPP (1 nM) in the presence and absence of TSA (100 nM), SAHA (1 µM), and VPA (2 mM) for 72 h at 37°C. Additionally, for PD-1 blockade, anti-PD1 antibody (3 µg/ml; BioLegend, San Diego, CA, USA) was added along with HDAC inhibitors. These effectors were then cocultured with zoledronate treated tumor targets (AW13516 Oral cancer cell line, COLO-205 Colon cancer cell line and Raji B lymphoblastic cell line) for 4 h in polypropylene tubes (BD Biosciences, USA) at effector target ratio of 4:1 in presence of monensin (5 mg/ml; Sigma-Aldrich) as described previously (36). Anti CD107a APC Ab (BioLegend, San Diego, CA, USA) was added at the start of coculture assay. After 4 h, cells were washed, fixed, and stained with anti-human TCR γδ PE and Granzyme B-PECF-594 (BD Biosciences, USA). Cells were acquired on FACS Aria (BD Biosciences, USA) and analysis was done by using FlowJo software (Tree Star, Ashland, OR, USA). The expression of various cell surface markers and intracellular proteins were analyzed on the γδ TCR^+^ cells gated populations.

For cell cycle analysis, 1 × 10^6^ γδ T cells treated with HDAC inhibitors VPA (0.5, 1, 2 mM), TSA (25, 50, 100 nM), and SAHA (0.25, 0.5, 1 µM) for 72 h or kept untreated, were harvested, and fixed by adding chilled 70% ethanol. Next day, cells were washed with PBS and stained with DNA intercalating dye propidium iodide (PI) along with RNAse A at a concentration of 40 and 10 µg/ml, respectively. Cells were incubated at room temperature for 30 min. The samples were acquired on FACS Calibur (BD Biosciences, USA) and analyzed using ModFit software.

### Proliferation Assay

Proliferation of γδ T cells was analyzed using ^3^H-Thymidine (3HTdR) incorporation assay. A total of 5 × 10^4^ γδ T cells were treated in the presence or absence of HDAC inhibitors VPA (0.5, 1, 2 mM), TSA (25, 50, 100 nM), and SAHA (0.25, 0.5, 1 μM) along with HDMAPP (1 nM) and rIL2 (50 IU/ml) for 72 h in 96-well tissue culture plates. The cultures were pulsed with 1 μCi [^3^H] thymidine (Board of Radiation and Isotope Technology, Mumbai) 18 h prior to termination of the assay. Following the incubation, cells were transferred upon glass-wool filters using cell harvester (Perkin Elmer, UK). The radioactivity incorporated into the DNA was measured in a liquid beta scintillation counter (Packard, Meriden, CT, USA). Data were expressed as counts per minute (cpm).

### Cytokine ELISA

For cytokine ELISA, supernatants were collected from γδ T cells treated in the presence of different concentrations of HDAC inhibitors VPA (0.5, 1, 2 mM), TSA (25, 50, 100 nM), and SAHA (0.25, 0.5, 1 μM) along with HDMAPP (1 nM) and rIL2 (50 IU/ml) for 24 h. The concentration of secreted cytokines IFNγ and TNFα was measured by human IFN-γ and TNF-α ELISA kit, respectively (BD Biosciences, USA) as per manufacture’s procedure.

### Cytotoxicity Assay

Cytotoxic potential of γδ T cells against panel of tumor cell lines, oral tumor cell line (AW13516), colon tumor cell line (COLO-205), and B lymphoblastic cell line (Raji) was performed using lactate dehydrogenase (LDH) release assay as described previously (37). Tumor cell lines were treated for 18 h with zoledronate (100 µM; Sigma-Aldrich). γδ T cells were treated with HDMAPP (1 nM) and rIL-2 (50 IU/ml) in presence and absence of HDAC inhibitors, VPA (2 mM), TSA (100 nM), and SAHA (1 µM) for 72 h at 37°C were used as effectors. Additionally, for PD-1 blockade, anti-PD1 antibody (3 µg/ml) was added to HDAC inhibitor treated γδ T cells for 72 h at 37°C and were also used as effectors. Briefly, tumor cell lines were cocultured with effectors at 40:1 effector target (E/T) ratio for 4 h at 37°C in 96-well plates (Nunc, Denmark). After 4 h of coculture, an aliquot of 50 µl of media was used in LDH cytotoxic assay using the LDH cytotoxic assay kit (Thermo Fisher Scientific, USA) according to manufactures protocol. γδ T cell cytotoxicity was defined as % specific lysis = Experimental value – Effector cells spontaneous control − Target cells spontaneous control/Target Cell Maximum Control − Target cells spontaneous control.

### Chromatin Immunoprecipitation (ChIP) qPCR Assays

Chromatin Immunoprecipitation assays were performed using MAGnify TM Chromatin Immunoprecipitation System (Thermo Fisher Scientific, USA) according to the manufacturer’s instructions. Specific anti-acetyl histone H3 (Abcam) and anti-acetyl histone H4 (Abcam) were used to determine the promoter acetylation of perforin and granzyme-B. Normal rabbit IgG was used as negative control. DNA was extracted and analyzed by quantitative real time PCR (qPCR) with specific primers for perforin (region I forward:5′-GATGAGGGCTGAGGACAG-3′; region I reverse:5′-TCTTCACCGAGGCTCCTG-3′; region II forward:5′-CTGCTGGCCTGTTCATCAAC-3′; region II reverse: 5′-CTGTCCTCAGCCCTCATC-3′) and granzyme B (region I forward: 5′-GGGTGGGCAGCATTTACAG-3′; region I reverse: 5′-TTCTCAGGAAGGCTGCCC-3′; region II forward: 5′-CACTTCATAGGCTTGGGTTCC-3′; region II reverse: 5′-CCTCTGGTTTTGTGGTGTCTC-3′). 1% of starting chromatin was used as input. Relative data quantification was performed using 2^−ΔΔCt^ method, using formula: % Input = 2 (Ct Input − Ct ChIP) × Input dilution factor × 100 and expressed in the form of % input as described earlier (38).

### Statistical Analysis

Data analysis was done by Student’s *t*-test using GraphPad Prism software (GraphPad Software Inc., CA, USA). The comparative CT method was applied in the quantitative real time RT-PCR according to 2^−(ΔΔCt)^ method. Results were indicated as means ± SE and considered significant at *p* < 0.05.

## Results

### Effect of HDAC Inhibitors on Viability of γδ T Cells

We first studied effects of HDAC inhibitors VPA (0.25–4 mM), TSA (25—200 nM), and SAHA (0.25–4 μM) on the viability of γδ T cells. Magnetically sorted γδ T cells from peripheral blood of healthy individuals were activated with HDMAPP (1 nM) and rIL2 (50 IU/ml) in presence and absence of above mentioned HDAC inhibitor concentrations for 72 h. HDMAPP is a synthetic analog of IPP and potent activator of γδ T cells. Immunomagnetically sorted γδ T cells were positive for γδ TCR (90%), CD56 (53%), and negative for αβ TCR, CD14, and CD 19 (Figure S2A in Supplementary Material). Viability of γδ T cells was assessed by MTT assay. It was observed that higher concentrations of HDAC inhibitors were toxic to γδ T cells. γδ T cells showed least viability at VPA (3–4 mM); TSA (150–200 nM), and SAHA (3–4 μM). At lower concentrations, these HDAC inhibitors were not toxic and γδ T cell were viable (>90%) (Figure S1 in Supplementary Material). For further validation of viability, γδ T cells activated with HDMAPP and rIL2 in the presence or absence of HDAC inhibitorsVPA (0.5–2 mM), TSA (25–100 nM), and SAHA (0.25–1 μM) were stained with Annexin V and 7-AAD. We observed that at these concentrations HDAC inhibitors did not induce any significant apoptosis. Since HDAC inhibitor concentrations, VPA (0.5–2 mM), TSA (25–100 nM), and SAHA (0.25–1 μM) showed least effect on the viability of γδ T cells (Figures S2B,C in Supplementary Material), these were selected in further experiments.

### HDAC Inhibitors Inhibit the Antigen-Driven Proliferation and Cell Cycle Progression of γδ T Cells

**Figure 1:**
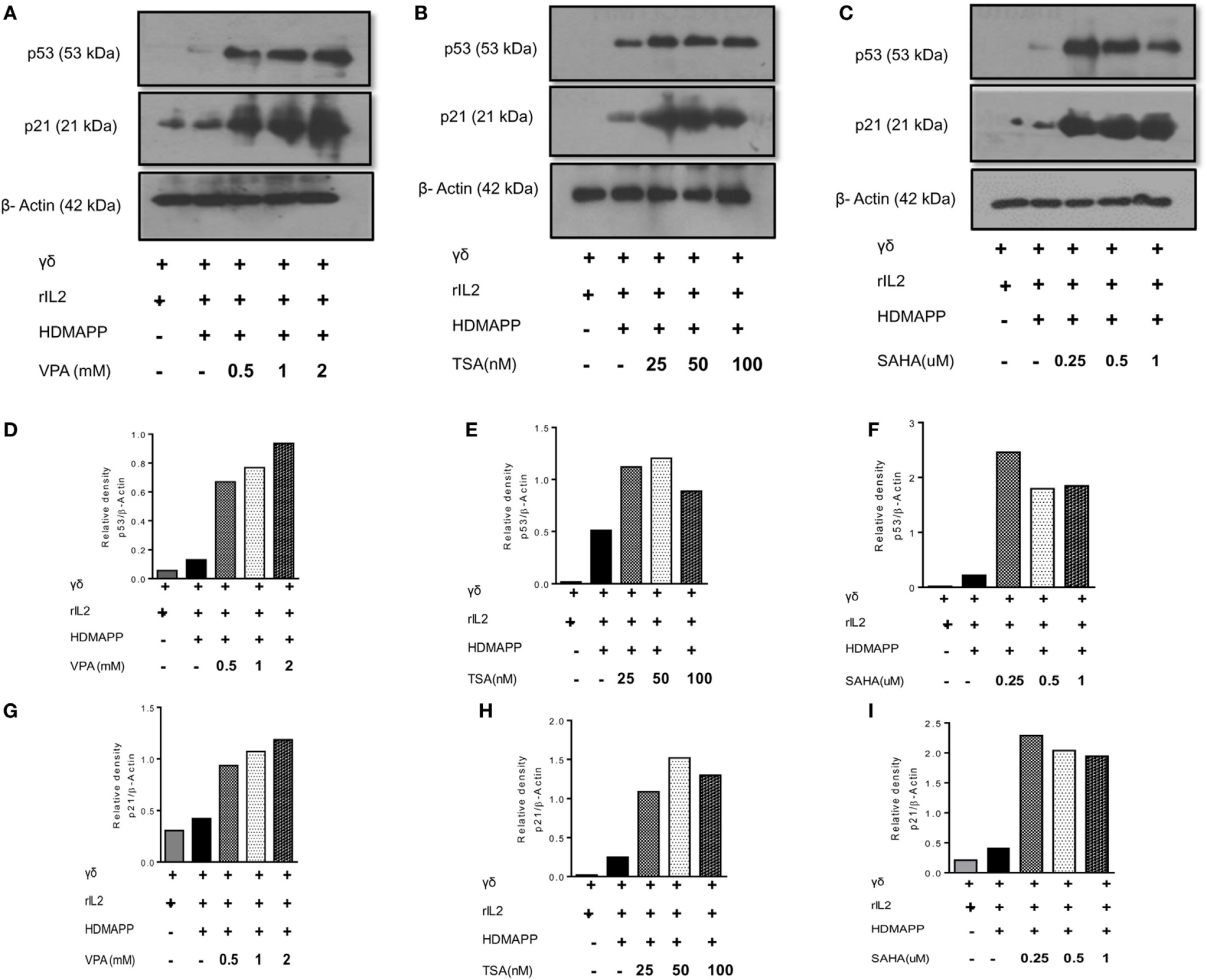
Histone deacetylases (HDAC) inhibitors increase the expression of cell cycle checkpoint proteins p53 and p21. Protein expression of p53 and p21 by γδ T cells upon treatment with **(A)** sodium valproate (VPA), **(B)** Trichostatin-A (TSA), and **(C)** suberoylanilidehydroxamic acid (SAHA) as detected by western blotting. Cell lysates of γδ T cells, stimulated with HDMAPP after treatment with HDAC inhibitors at different concentrations for 72 h were prepared and p53, p21 proteins were detected. β-actin was used as loading control. Densitometry quantification of p53 **(D–F)** and p21 **(G–I)** expression in γδ T cells upon treatment with VPA, TSA, and SAHA, relative to β-actin.

γδ T cell show robust proliferation when stimulated with phosphoantigen (HDMAPP) in presence of rIL2. In order to investigate the effect of HDAC inhibitors on proliferation of γδ T cells, γδ T cells were stimulated with phosphoantigen HDMAPP and rIL2 in the presence or absence of different concentration of HDAC inhibitors (VPA; 0.5–2 mM, TSA; 25–100 nM, and SAHA; 0.25–1 μM) and proliferation was monitored using ^3^H thymidine incorporation assay. γδ T cells showed robust proliferative responses to phosphoantigen HDMAPP in presence of rIL-2, compared to unstimulated γδ T cells. However, in the presence of various concentrations of VPA, TSA, and SAHA, the proliferative responses of γδ T cells were significantly reduced in a concentration-dependent manner (Figure S3A in Supplementary Material), with maximum decrease in proliferation of γδ T cells observed at higher concentration of HDAC inhibitors, VPA 2 mM, TSA 100 nM, and SAHA 1 µM, respectively. Further, we also evaluated the role of HDAC inhibitors on cell cycle progression of γδ T cells. Freshly isolated γδ T cells were stimulated with HDMAPP and rIL2 in presence or absence of different concentrations of HDAC inhibitors. Upon stimulation with HDMAPP and rIL2, significant number of γδ T cells were in S phase and G2/M phase. However, upon treatment of HDAC inhibitors, γδ T cells were arrested in G0/G1 phase (Figures S3B,C in Supplementary Material). This inhibition of cell cycle progression in γδ T cells upon HDAC inhibitor treatment was reflected in the increased expression of p53 and its downstream target p21, suggesting that HDAC inhibitors impede the G0/G1-S phase transition in γδ T cells in p53-dependent manner (Figures 1A–C) and (Figures 1D–I).

### HDAC Inhibitors Regulate Cytokine Production and Activation in γδ T Cells

**Figure 2:**
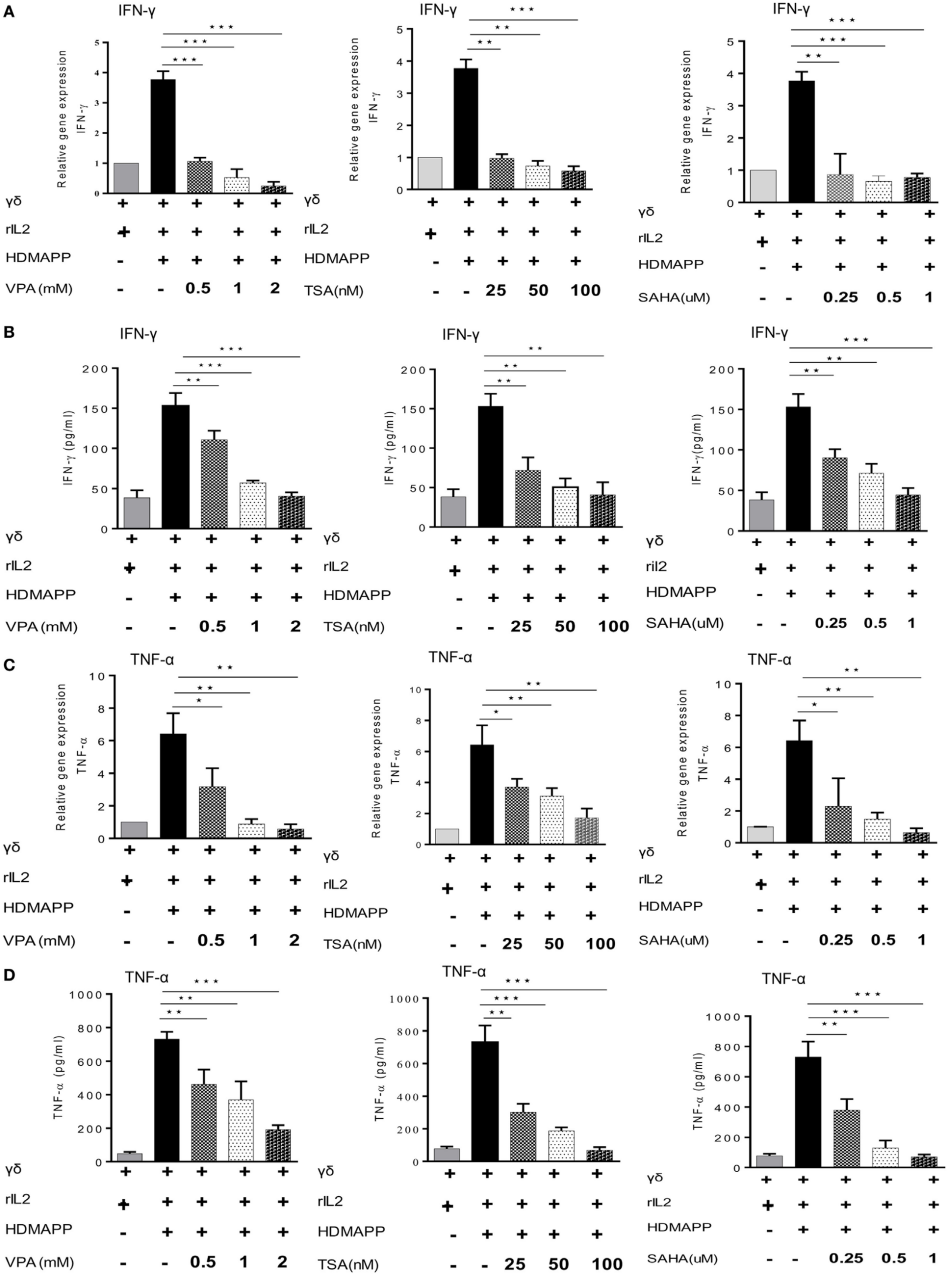
Histone deacetylases (HDAC) inhibitors regulate cytokine production. Expressions of IFN-γ and TNF-α were detected by quantitative real-time PCR and sandwich ELISA. **(A,B)** IFN-γ expression by γδ T cells stimulated with HDMAPP, treated with or without HDAC inhibitors sodium valproate (VPA), Trichostatin-A (TSA), and suberoylanilidehydroxamic acid (SAHA) at different concentrations at mRNA and protein levels, respectively. **(C,D)** Expression of TNF-α in the supernatants collected from HDMAPP stimulated γδ T cells in the presence or absence of HDAC inhibitors VPA, TSA, and SAHA at different concentrations at mRNA and protein levels, respectively. The expression of different m-RNA transcripts was normalized to 18S r-RNA. All the results indicated are mean ± SEM of three independent experiments, where *p < 0.05, **p < 0.005, ***p < 0.0005.

**Figure 3:**
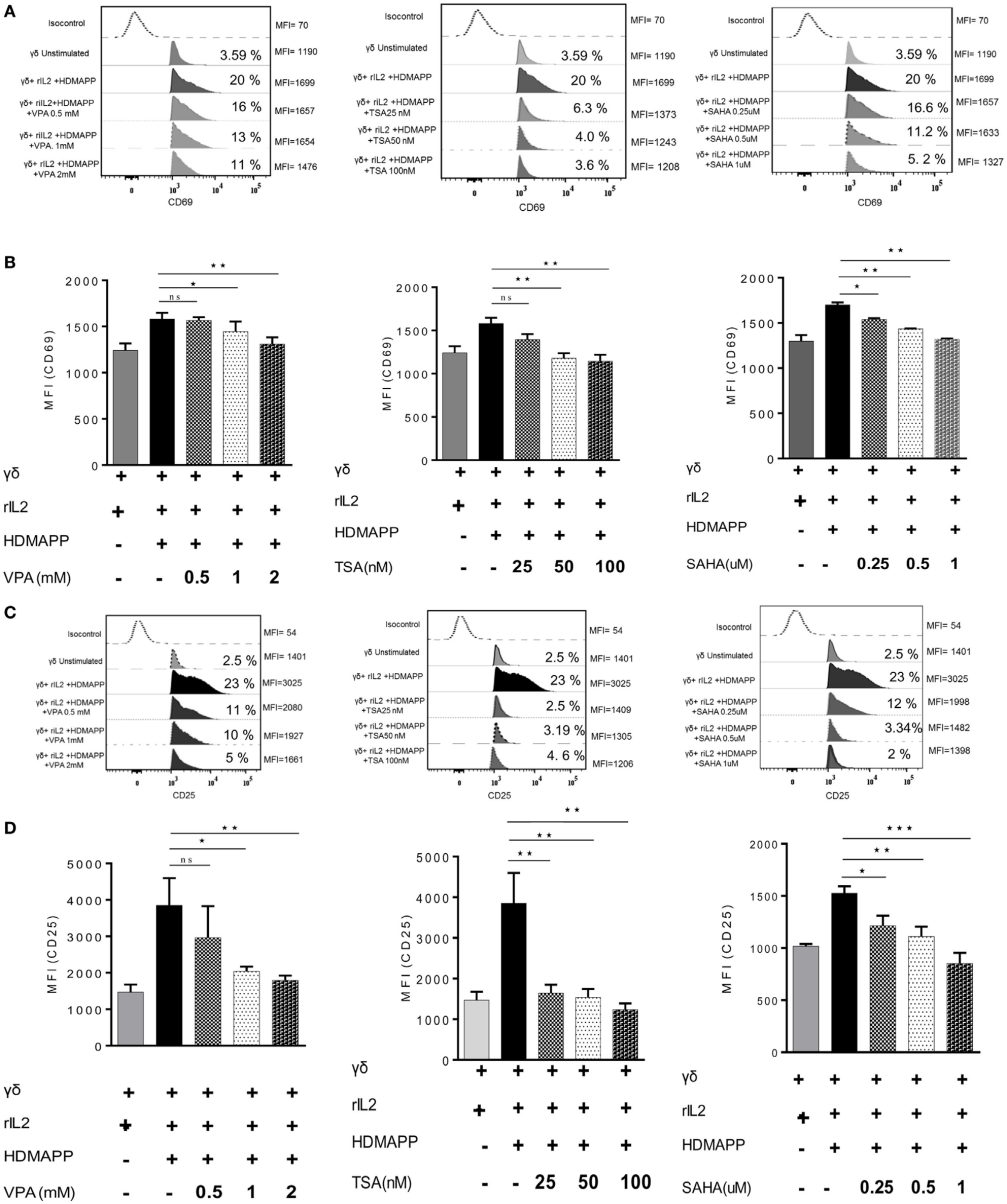
Histone deacetylases (HDAC) inhibitors affect the activation markers on γδ T cells. **(A)** The expression of early activation marker (CD69) on unstimulated γδ T cells and HDMAPP and rIL-2 stimulated γδ T cells with or without HDAC inhibitor treatment was analyzed by multi-color flow cytometry. Values on right side indicate the median fluorescence intensity (MFI) of CD69, while the values inside the histogram represent the percent CD69-positive γδ T cells. The histograms shown are representative of three independent experiments. **(B)** The cumulative MFI of CD69 expression on γδ T cells is represented as bar graphs. Data shown are representative of three independent experiments where *p < 0.05, **p < 0.005, ***p < 0.0005. **(C)** The effect of HDAC inhibitors on expression of late activation marker CD25 was assessed by flow cytometry. Unstimulated γδ T cells and HDMAPP stimulated γδ T cells with or without HDAC inhibitors at different concentrations, after 72 h were stained with the flurophore-tagged antibody and acquired on FACS Aria. Values on right side indicate the MFI of CD25, while the values inside the histogram represent the percent CD25-positive γδ T cells. The histograms depicted are representative of three independent experiments. **(D)** The results shown are cumulative MFI of CD25 expression on γδ T cells. HDAC inhibition decreases expression of CD25. Data shown are representative of three independent experiments where *p < 0.05, **p < 0.005, ***p < 0.0005.

γδ T cells upon activation secrete copious amount of cytokines such as IFN-γ and TNF-α (36). We examined the effect of HDAC inhibitors on expression of these cytokines in γδ T cells. Marked increase in the expression of cytokines IFN-γ and TNF-α was observed upon stimulation of γδ T cells with HDMAPP and rIL2 compared to unstimulated γδ T cells. Expression of IFN-γ (Figures 2A,B) and TNF-α (Figures 2C,D) was decreased significantly when treated with HDAC inhibitors TSA, SAHA, and VPA. This inhibition was observed both at mRNA and protein levels. It was observed that inhibition of cytokine expression was concentration dependent for HDAC inhibitors. We also evaluated the effect of HDAC inhibitors on the expression of early activation marker CD69 and late activation marker CD25 on γδ T cells. Treatment of γδ T cells with HDAC inhibitors led to decrease in the expression of early activation (Figures 3A,B) and late activation marker on γδ T cells (Figures 3C,D). The expression of these activation markers on γδ T cells were significantly reduced in a concentration-dependent manner, with maximum decrease at VPA 2 mM, TSA 100 nM, and SAHA 1 µM, respectively. Percentage of γδ T cells positive for these markers was also less in HDAC inhibitor treated γδ T cells as compared to untreated γδ T cells. To investigate the role of HDAC inhibitors on the expression of other activating receptors like NKG2D, CD16, and inhibitory receptors like KIR2DL2/3 (CD158b), γδ T cells were treated with HDAC inhibitors VPA 2 mM, TSA 100 nM, and SAHA 1 µM for 72 h. We found that HDAC inhibitor-treated γδ T cells show decreased expression of NKG2D (Figure S4A in Supplementary Material) as compared to untreated γδ T cells. On the contrary, γδ T cells treated with HDAC inhibitors VPA 2 mM, TSA 100 nM, and SAHA 1 µM show increase in the expression of inhibitory receptor KIR2DL2/3 (CD158b) (Figure S4B in Supplementary Material). However, we did not observe any change in CD16 expression (Figure S4C in Supplementary Material). Collectively, the data advocate the role of HDAC inhibitors in abating the expression of activation markers (CD69, CD25, NKG2D) and cytokine (IFN-γ, TNF-α) production in γδ T cells.

### HDAC Inhibitors Suppress the Expression of Transcription Factors Eomes and Tbet in γδ T Cells

**Figure 4:**
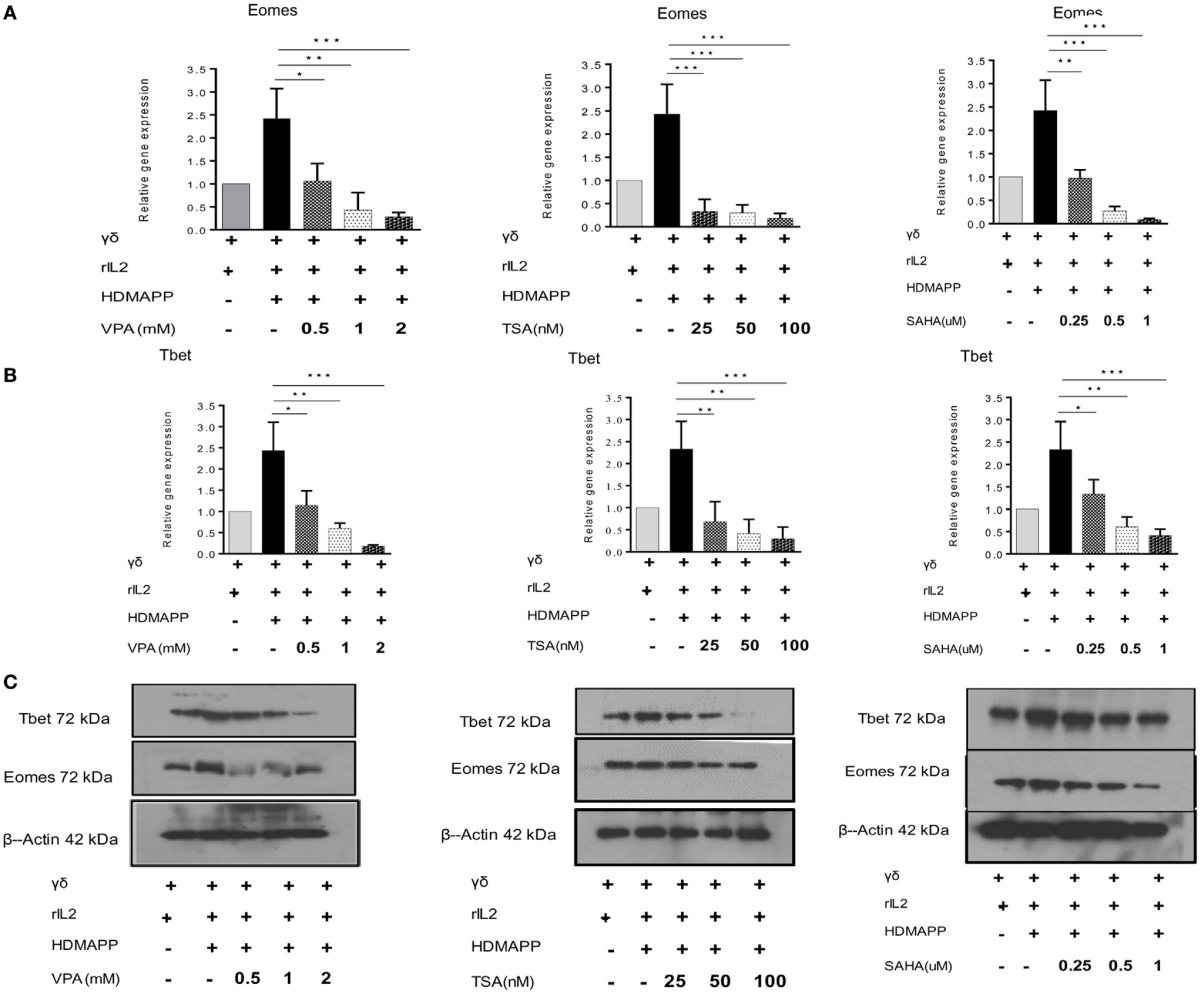
Histone deacetylases (HDAC) inhibition abrogates expression of transcription factors regulating effector functions of γδ T cells. The m-RNA expression of Eomes **(A)** and T bet **(B)** in γδ T cells activated with HDMAPP, in the presence or absence of HDAC inhibitors sodium valproate, Trichostatin-A, and suberoylanilidehydroxamic acid at different concentrations was quantified by quantitative real-time PCR. The results indicated are cumulative mean of relative gene expression normalized to 18S r-RNA where *p < 0.05, **p < 0.005, ***p < 0.0005, compared with γδ T cells activated with HDMAPP. The data shown are representative of three independent experiments. **(C)** The protein level expression of T bet and Eomes was detected by western blotting. HDAC inhibitor treatment decreases the expression of T-bet and Eomes. β-actin was maintained as loading control. The blots shown are representative of three experiments.

Eomes and Tbet are two main T-box transcription factors expressed in T cells. They are the main transcription factors, which regulate the effector functions of CD8 T cells through the expression of effector genes perforin and granzyme B (39, 40). Besides CD8 T cells, γδ T cells also express Eomes and Tbet (41). Upon activation with phosphoantigen (HDMAPP) and rIL2, γδ T cells show increased expression of these two transcription factors. We hypothesized that HDAC inhibitors may have an impact on the expression of these two transcription factors in γδ T cells. Therefore, the role of HDAC inhibitors was analyzed in regulating expression of Eomes and Tbet in γδ T cells activated with phosphoantigen (HDMAPP) and rIL2. γδ T cells treated with HDAC inhibitors showed decrease in the expression of Eomes and Tbet at both mRNA (Figures 4A,B) and protein level (Figure 4C). In addition to Eomes and Tbet transcriptional factors, γδ T cells treated with HDAC inhibitors also show decreased expression of NF-κB (Figures S4D–F in Supplementary Material) as compared to untreated γδ T cells. Inhibition of Eomes, Tbet, and NF-κB by HDAC inhibitors clearly demonstrates that HDAC inhibitors regulate the effector functions of γδ T cells.

### HDAC Inhibitors Inhibit the Antitumor Cytotoxic Potential of γδ T Cells

**Figure 5:**
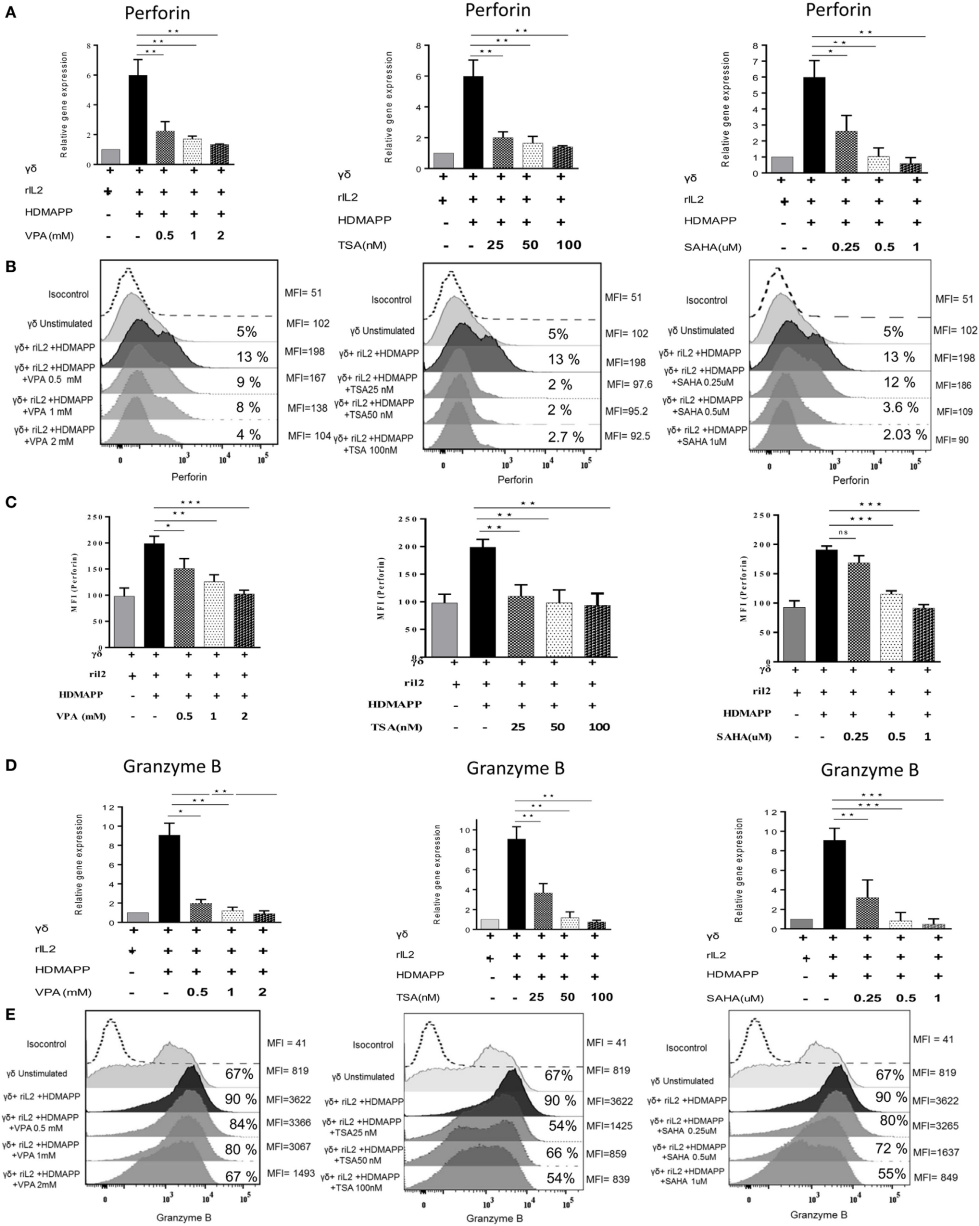

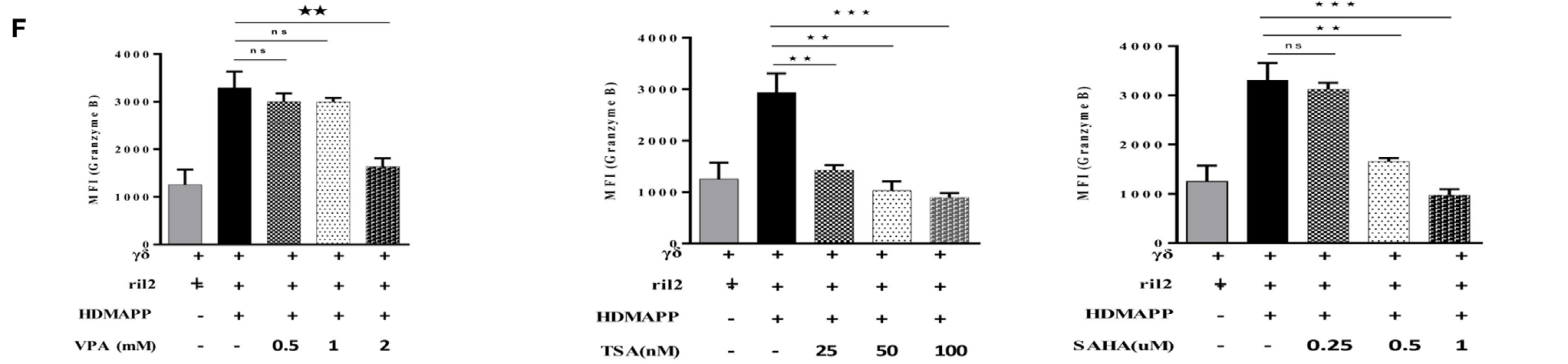
Histone deacetylases (HDAC) inhibitor treatment abrogates the antitumor potential of γδ T cells. Expression of perforin and granzyme was studied at mRNA and protein levels using quantitative real-time PCR and flow cytometry, respectively. The gene expression of **(A)** perforin and **(D)** granzyme B by HDMAPP-stimulated γδ T cells with or without HDAC inhibitor treatment was quantified by quantitative real-time PCR. The results shown are cumulative means of relative gene expression, normalized to 18S r-RNA, and representative of three independent experiments. **(B)** γδ T cells activated by HDMAPP with or without sodium valproate, Trichostatin-A, and suberoylanilidehydroxamic acid at different concentrations were stained after 72 h with corresponding flurophore tagged antibody and expression of perforin was analyzed by flow cytometry. The values on right side of histograms indicate median fluorescence intensity (MFI) of perforin, while the values inside the histogram represent the percent positive γδ T for perforin. **(C)** Bar graphs represent cumulative MFI values where *p < 0.05, **p < 0.005, ***p < 0.0005, compared with γδ T cells activated with HDMAPP. **(E)** Expression of granzyme B by unstimulated γδ T cells and HDMAPP activated γδ T cells with or without HDAC inhibitor treatment was assessed by flow cytometric analysis. The values on right side of histograms indicate MFI of granzyme B, while the values inside the histogram represent the percent positive γδ T for granzyme B. The histograms are representative of three independent experiments. **(F)** The graphs represent MFI values of granzyme B where *p < 0.05, **p < 0.005, ***p < 0.0005.

**Figure 6:**
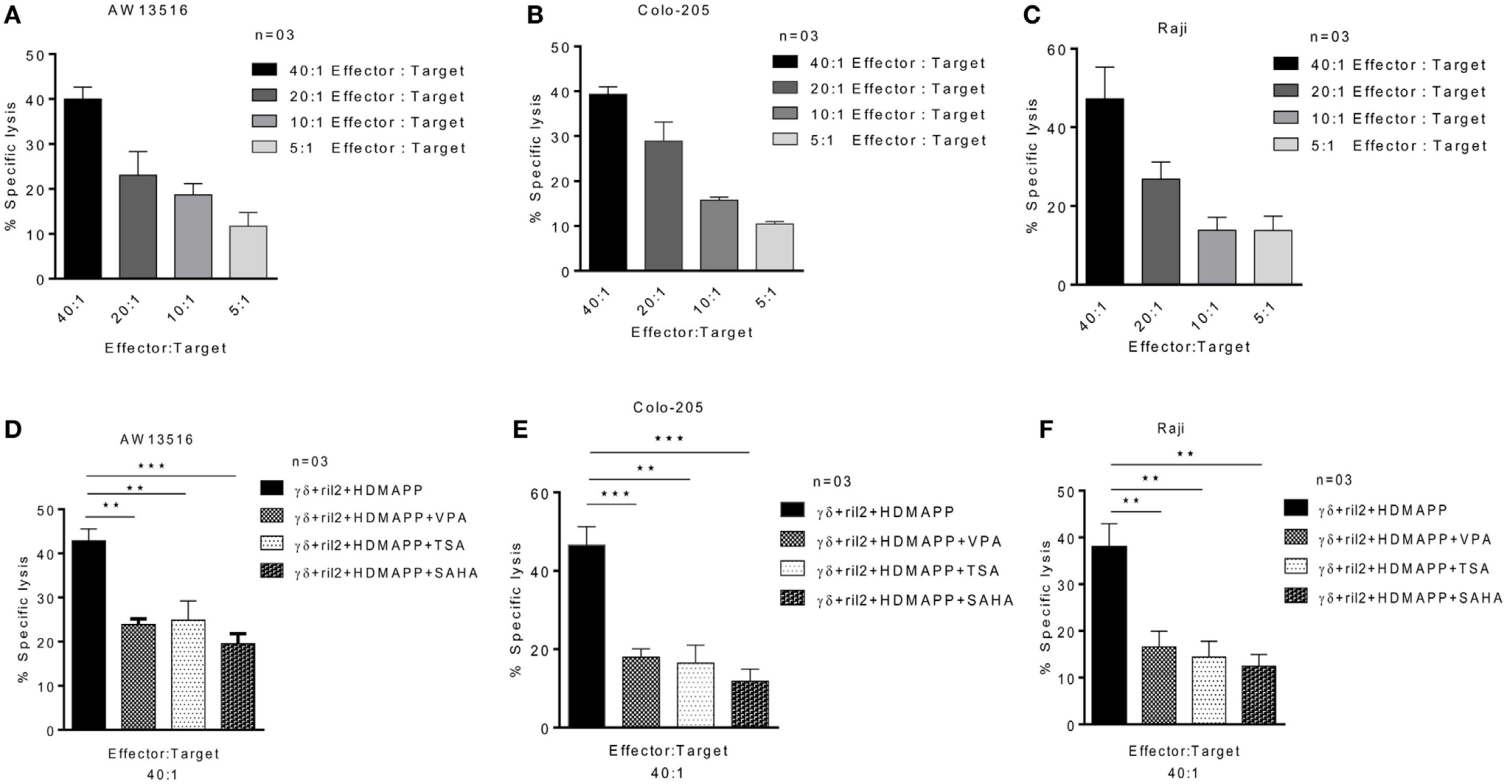
Histone deacetylases inhibitors decrease the cytotoxic effector functions of γδ T cells. Cytotoxic effector function of γδ T cells was assessed against three tumor cell lines **(A)** AW13516, **(B)** COLO205, and **(C)** Raji. Zoledronate-treated tumor targets were cocultured with γδ T cells for 4 h and cytotoxicity was assessed by lactate dehydrogenase (LDH) assay. The results indicated are mean percent specific lysis at different effector:target ratio. Data are representative of three individual experiments. Cytotoxic potential of γδ T cells activated with HDMAPP in the presence of rIL-2 with or without treatment with sodium valproate (2 mM), suberoylanilidehydroxamic acid (1 µM), and Trichostatin-A (100 nM) was assessed against three zoledronate-treated tumor targets AW13516 **(D)**, COLO-205 **(E)**, and Raji **(F)** by LDH cytotoxicity assay. The results indicated are percent specific lysis at the effector to target ratio of 40:1 where **p < 0.005 and ***p < 0.0005 when compared with γδ T cells activated with HDMAPP in the presence of rIL-2 (*n* = 3).

To evaluate the role of HDAC inhibitors in modulation of antitumor potential of γδ T cells, we analyzed the expression of effector molecules Perforin and Granzyme B in γδ T cells at mRNA and protein level. Perforin and Granzyme B are the effector molecules, which are responsible for the antitumor functions of CD8 and γδ T cells (42, 43). Freshly isolated γδ T cells activated with phosphoantigen HDMAPP and rIL2 show increased expression of these two effector genes; however, γδ T cells activated in presence of HDAC inhibitors showed decrease in the expression of perforin (Figures 5A–C) and granzyme B. (Figures 5D–F). Maximum effect on the expression of perforin and granzyme B was observed with VPA 2 mM, TSA 100 nM, and SAHA 1 µM. These concentrations of HDAC inhibitors were used in further cytotoxicity experiments. We next evaluated whether decrease in expression of effector molecules perforin and granzyme B are regulated by histone modifications in γδ T cells. To investigate this, we checked the total histone H3 and H4 acetylation in γδ T cells treated with HDAC inhibitors VPA 2 mM, TSA 100 nM, and SAHA 1 µM. We observed that the total level of H3 acetylation and H4 acetylation increases in γδ T cells after treatment of HDAC inhibitors as compared to untreated γδ T cells (Figure S5A in Supplementary Material). However, HDAC inhibitor-treated γδ T cells show less histone H3 acetylation and H4 acetylation on promoters of perforin and granzyme B compared to untreated γδ T cells determined by ChIP qPCR assay. Histone acetylation is positively correlated with transcriptional activity. Thus, our data show that epigenetic changes on promoters of effector molecules perforin and granzyme B control the expression of these molecules in HDAC inhibitor treated γδ T cells (Figures S5B,C in Supplementary Material). The cytotoxic potential of HDAC inhibitor treated γδ T cells was evaluated against panel of zoledronate-treated tumor cells lines (AW13516, COLO-205, and Raji). At different E/T ratios starting from 5:1 to 40:1, HDMAPP-activated γδ T cells in the presence of IL-2 efficiently lysed zoledronate-treated tumor cells lines (AW13516, COLO-205, and Raji). Maximum cytotoxicity of γδ T cells was observed at E/T ratio of 40:1 (Figures 6A–C). This ratio of E: T was used in further experiments, to assess the effect of HDAC inhibitors TSA, VPA, and SAHA on cytolytic ability of γδ T cells. γδ T cells stimulated with HDMAPP and rIL2 in presence of HDAC inhibitors VPA (2 mM), TSA (100 nM), and SAHA (1 µM) for 72 h were used as effector against zoledronate-treated tumor cell lines (AW13516, COLO205 and Raji) as targets at E/T ratio of 40:1. Zoledronate, an aminobisphonate drug, inhibits the enzyme farnesyl pyrophosphate synthase in the mevalonate pathway leading to accumulation of IPP, which stimulates γδ T cell activation *via* TCR signaling. γδ T cells treated with different HDAC inhibitors showed significant decrease in their cytotoxic potential against zoledronate treated tumor targets (AW13516, COLO 205, and Raji) (Figures 6D–F). It was observed that all the three HDAC inhibitors (VPA, TSA, and SAHA) significantly inhibited the ability of γδ T cells to kill zoledronate treated tumor cell lines.

### HDAC Inhibitors Abrogate the Effector Functions of γδ T Cells by Upregulating the Immune Checkpoint Proteins PD-1 and PD-L1

**Figure 7:**
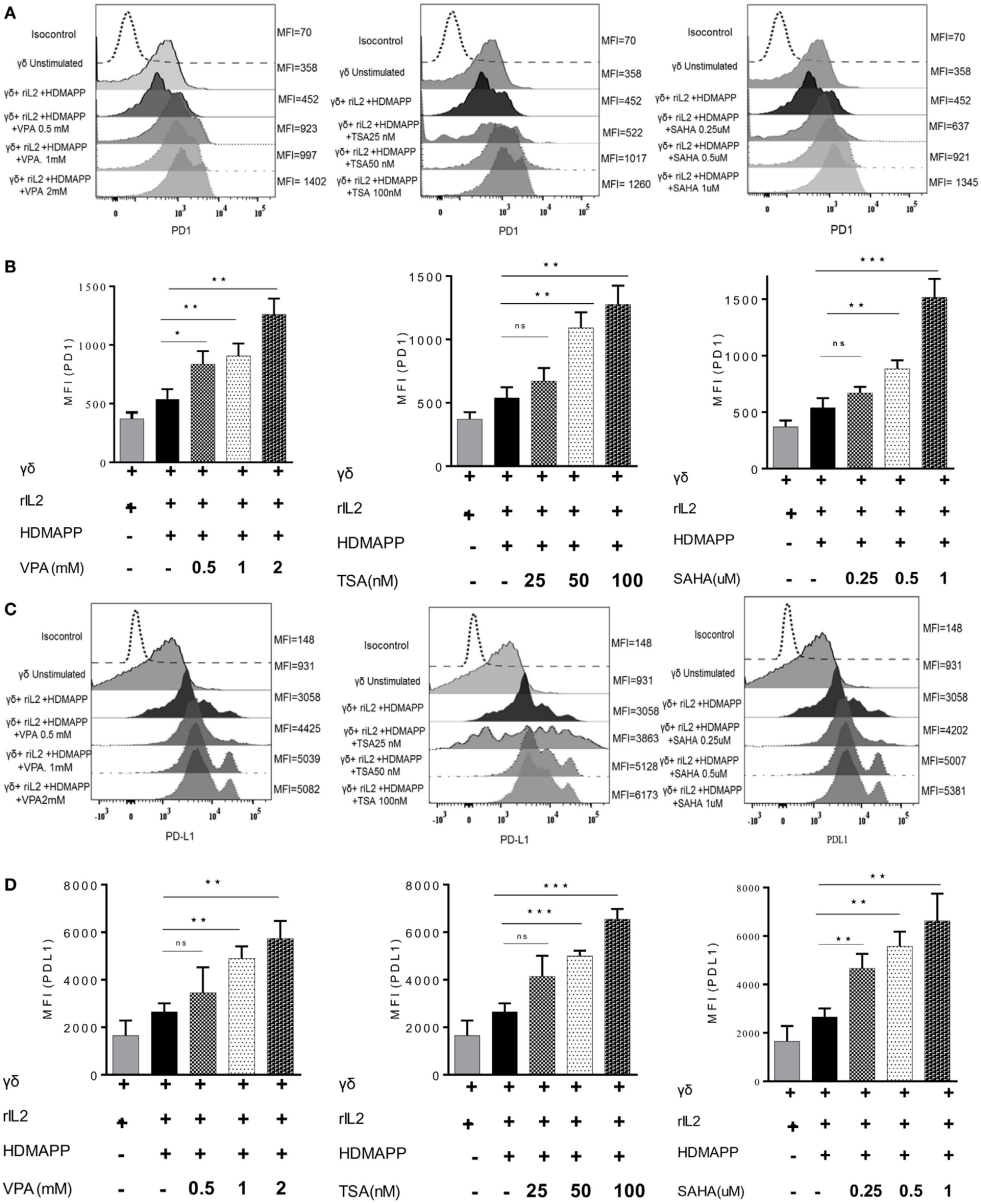
Histone deacetylases (HDAC) inhibitors upregulate the expression of immune checkpoints on γδ T cells. **(A)** The expression of programmed death-1 by HDAC inhibitor-treated γδ T cells at their respective concentration. Histograms are representative of three individual experiments. The values on right side of histograms indicate median fluorescence intensity (MFI) of PD1. **(B)** MFI of PD1 expression as bar graphs where *p < 0.05, **p < 0.005, ***p < 0.0005 and ns, not significant when compared with γδ T cells activated with HDMAPP in the presence of rIL-2. **(C)** The expression of programmed death ligand-1 (PD-L1) by γδ T cells treated with HDAC inhibitors sodium valproate, Trichostatin-A, and suberoylanilidehydroxamic acid at their respective concentration was analyzed by immunostaining. Histograms shown are representative of three individual experiments. The values on right side of histograms indicate MFI of PD-L1. The results indicated in **(D)** are MFI of PD-L1 expression where *p < 0.05, **p < 0.005, ***p < 0.0005 and ns, not significant when compared with γδ T cells activated with HDMAPP.

**Figure 8:**
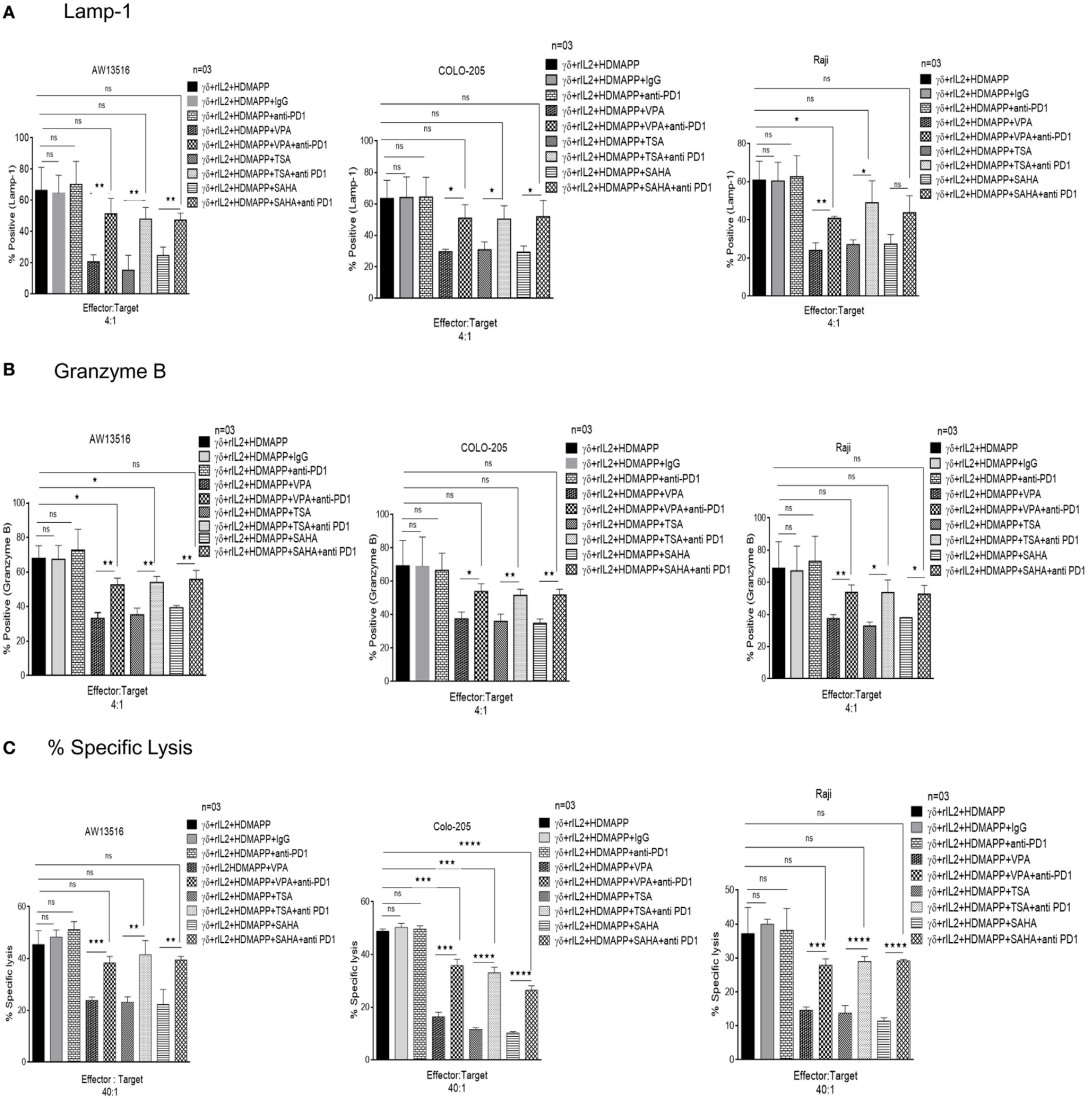
Histone deacetylases (HDAC) inhibitors abrogate effector functions of γδ T cells *via* programmed death-1 (PD-1) upregulation. Expression of of **(A)** degranulation marker CD107a and **(B)** granzyme B by γδ T cells was assessed by flow cytometric analysis. HDMAPP activated γδ T cells upon treatment with sodium valproate (VPA) (2 mM), Trichostatin-A (TSA) (100 nM), and suberoylanilidehydroxamic acid (SAHA) (1 µM) in the presence or absence of PD1 blocking antibody were cocultured with three zoledronate treated tumor targets (AW 13516, Raji, COLO205) cells for 4 h at effector to target ratio of 4:1. The data represent consolidated median fluorescence intensity values of granzyme B and CD 107a expressing cells, indicative of three independent experiments (**p < 0.005, *p < 0.05, and ns, not significant). **(C)** The cytotoxic ability of γδ T cells treated with HDAC inhibitors TSA, SAHA, and VPA in the presence or absence of PD-1 blocking antibody was assessed against three zoledronate treated tumor targets (AW 13516, Raji, COLO205) by lactate dehydrogenase cytotoxicity assay. HDAC inhibitor treated γδ T cells show increased cytotoxic potential in the presence of PD-1 blocking antibody. The results indicate percent cytotoxicity where **p < 0.005, ***p < 0.0005, and ns, not significant, when compared with HDMAPP-activated γδ T cells treated with the respective HDAC inhibitor. Data represent three independent experiments.

Programmed death-1 receptor and its ligand PD-L1 are commonly expressed on immune cells. PD-1 and PD-L1 belong to the family of immune checkpoint proteins that act as co-inhibitory signaling inducers. Upon activation, T cells show enhanced expression of immune check point PD-1. Interaction between PD-1 and PD-L1 halt the T cell activation, thus maintaining the immune homeostasis. Tumor cells exploit this pathway to evade immune response. The effect of HDAC inhibitors on the expression of PD-1 and PD-L1 on γδ T cells was studied. γδ T cells were treated with different concentrations of HDAC inhibitors and expression of PD-1 and PD-L1 was analyzed by flow cytometry. Upon activation with antigen HDMAPP and rIL-2, expression of PD-1 and PD-L1 increases on γδ T cells. However, the expression of PD-1 and PD-L1 on γδ T cells substantially increased upon treatment with HDAC inhibitors. Maximum increase in the expression of PD-1 (Figures 7A,B) and PD-L1 (Figures 7C,D) on HDMAPP and rIL-2 activated γδ T cells was observed after treatment with VPA (2 mM), TSA (100 nm), and SAHA (1 µM). To assess the role of PD1/PD-L1 signaling in HDAC inhibitor treated γδ T cells, γδ T cells were activated with HDMAPP and rIL2, treated or untreated with HDAC inhibitors for 72 h. PD-1 blocking antibody was added at the start of culture. After 72 h, these γδ T cells were cultured with zoledronate-treated tumor cell lines AW13516, COLO-205, and Raji for 4 h at E/T ratio of 4:1. Blockade of PD-1 in HDAC inhibitor treated HDMAPP activated γδ T cells rescued the expression of effector molecules Lamp-1 (CD107a) (Figure 8A) and granzyme B (Figure 8B) as compared to only HDAC inhibitor treated γδ T cells. To further evaluate the role of HDAC inhibitors on the PD1/PD-L1 signaling axis in γδ T cells, we did the similar experiment by coculturing the effectors and above mentioned tumor targets to analyze the cytotoxic potential by LDH release assay at a ratio of 40:1 for 4 h. Blocking of PD-1 in HDMAPP-activated γδ T cells treated with HDAC inhibitorsVPA (2 mM), TSA (100 nM), and SAHA (1 µM) improves the cytolytic potential of γδ T cells as compared to γδ T cells treated with HDAC inhibitor only (Figure 8C). Thus, the results shows that blockade of PD-1 and PD-L1 signaling in HDAC inhibitor treated γδ T cells rescue their effector functions.

## Discussion

γδ T cell immunotherapy has become the emerging lead in the landscape of cancer immunotherapies due to their distinctive immune features and potent antitumor effector functions. They have been extensively targeted against diverse tumors such as melanoma, renal cell carcinoma, as well as B cell malignancies and have shown promising results in clinical settings (44). While these therapies have encountered modest clinical success, they have to overcome certain challenges such as limited availability of γδ T cells and rapid exhaustion upon repeated *in vitro* activation. Hence, combinational approaches have been envisaged with chemotherapeutics, monoclonal antibodies, small molecule inhibitors, etc. Newer treatment modality may include combining γδ T cell immunotherapy with antitumor drugs and other immune-modulating antibodies.

Epigenetic dysregulation is one of the hallmarks of cancer. Hence, epigenetic modifiers such as HDAC inhibitors are being comprehensively explored for their anticancer potential. Besides anticancer properties, HDAC inhibitors have also shown promising results in controlling the other pathological conditions such as neurological disorders and viral infections and are well tolerated (45, 46). Currently, VPA along with other short-chain fatty acids HDAC inhibitors are being clinically evaluated as anticancer drugs (47). HDAC inhibitors employ wide range of antitumor mechanisms such as induction of apoptosis, senescence, differentiation, or inhibition of cell cycle (48, 49). Vorinostat (SAHA), is among the first HDAC inhibitor to be approved by United States Food and Drug Administration (FDA) for the treatment of relapsed and refractory cutaneous T-cell lymphoma (50). Although HDAC inhibitors are approved for hematological malignancies, but clear proof-of-concept data for the clinical efficacy of HDAC inhibitors in solid tumors remains to be established (51). Recent studies have demonstrated that HDAC inhibitors exhibit higher therapeutic efficiency when combined with other antineoplastic agents (52). Hence, there is growing interest in exploring other combined therapeutic strategies with HDAC inhibitors.

Emerging evidence suggest that HDAC play a crucial role in T cell differentiation and effector functions. A number of studies have demonstrated that HDAC inhibitors suppress the immune response of T cells in severe inflammatory conditions and induce tolerance in organ transplantation (53). Specifically, HDAC inhibitors have shown to induce the regulatory T cell (Tregs) generation or stabilization of Tregs in inflammatory microenvironment due to which they have shown promising responses in experimental colitis (54). HDAC inhibitors increase the immunogenicity of tumors by increasing the expression of tumor antigens recognized by the immune cells. The antitumor responses of cytotoxic T lymphocytes like γδ T cells are mediated through recognition of stress molecules (ULBP, HSPs) or danger signals like MICA/B expressed on tumor cells by class of activating receptors known as NKG2D (55–57). Studies have demonstrated that HDAC inhibitors upregulate the NKG2D ligands on tumor cells, thereby sensitizing tumor cells to cytotoxicity mediated by γδ T cells in bladder cancer as well as NK cells in other malignancies such as osteosarcoma, pancreatic cancer, and multiple myeloma (32, 58–60). However, the causal effect of HDAC inhibitors on immune scenario is not well investigated and is contradictory. Several studies have shown that HDAC inhibitors affect each immune subset distinctly either leading to activation as in the case of CD4 T cells and CD8 T cells or by abrogating the effector functions of cells such as NK cell (61–63). Furthermore, for a particular immune cell type, the nature of immune regulation differs based on the type of HDAC inhibitor (64, 65). A recent study demonstrated that NKG2D expression in NK cells is inhibited by VPA (66).

Most of the studies have focused on investigating the impact of HDAC inhibitors on tumor cell lines and immune cells other than γδ T cells. Report by Suzuki et al. demonstrated that the antitumor effect of γδ T cells on bladder cancer was enhanced by treatment with VPA (32). The study focuses only on the impact of HDAC inhibitor, VPA on bladder cancer cell line. VPA leads to increase in the expression of MICA and MICB, which are recognized by NKG2D receptor on γδ T cells. The study does not explain the direct effect of HDAC inhibitors on γδ T cells. Earlier study by Kabelitz et al. reported that HDAC inhibitor VPA induces differential modulation of cell surface markers on γδ T cells compared to αβ T cells (67). Although the study shows the direct effect of VPA on γδ T cells, the functional responses of γδ T cells were not investigated in detail. In the present study, we have used three different HDAC inhibitors to delineate their effect on the functional responses of pure and sorted population of γδ T cells. We used clinically relevant concentrations of VPA, TSA, and SAHA in our study, which have been used in *in vitro* studies by other investigators (68, 69). We showed that three different HDAC inhibitors used suppressed the antitumor effector functions of γδ T cells.

We observed that γδ T cells activated with the phosphoantigen, HDMAPP in the presence of HDAC inhibitors showed decreased proliferative potential. One of the mechanism by which HDAC inhibitors exhibit their anticancer properties is through induction of cell differentiation and cell cycle arrest at G1 phase (48, 49). Besides affecting histone proteins, these inhibitors also have several non-histone protein substrates like p53, p21, Rb, and E2F1 in tumors (70, 71). On the other hand, it was demonstrated that downmodulation of p53 in T cells enhances their antigen-specific proliferative response and also augments antitumor cytotoxic functions (72, 73). Studies from our lab have shown that CD3-activated T cells upon activation show robust proliferative capacity and decreased expression of p53 and its downstream target p21 (74). Thus, the decrease in the antigen-specific proliferative response of γδ T cells in presence of HDAC inhibitors incited us to look for effect of HDAC inhibitors on cell cycle progression and expression of cell cycle regulators p53 and its downstream target p21. Decrease in the proliferation of γδ T cells in presence of HDAC inhibitors was associated with the increase in the expression of p53 and its downstream target p21. γδ T cells show increased expression of activation markers CD69 and CD25 when activated with phosphoantigens (36, 75). We observed that HDAC inhibitors inhibit the expression of CD69 and CD25 activation markers. CD25 is the high-affinity IL-2 receptor subunit and IL-2 signaling is necessary for the proliferation of T cells. It would be logical to conclude that HDAC inhibitors abrogate the IL-2 signaling and thus inhibit the proliferation of γδ T cells. We have used three different HDAC inhibitors VPA, TSA, and SAHA at different concentrations and they showed varied effects on expression of all the γδ T cell markers we studied. The likely explanation for the differences observed in their effects could be their structural diversity and also the biological activities they exert may be cell-type dependent.

Activated γδ T cells express Tbet and eomesodermin (Eomes) transcription factors. The T-box transcription factors T-bet and Eomes are important for acquisition of effector functions in cytotoxic T cells including γδ T cells (41, 76). Eomes and T-bet are highly homologous transcription factors and have cooperative and redundant functions in regulating the expression of different genes involved in the effector functions of CD8 T cells and activated natural killer cells. T-bet and Eomes regulate the expression of perforin, Granzyme-B, and IFN-γ by binding to promoter regions of these effector genes (14, 39). Knowing that HDAC inhibitors decrease the activation and proliferation of γδ T cells, we further hypothesized that HDAC inhibitors may modulate the effector functions of γδ T cells by affecting the expression of transcription factors Eomes and T-bet. We observed that treatment of γδ T cells with HDAC inhibitors lead to decrease in the expression of Eomes and T-bet. To further establish impact of HDAC inhibitors on the antitumor cytotoxic function of γδ T cell, we used panel of tumor cell lines (AW13516, COLO-205, and Raji) treated with zoledronate as target cell line in cytotoxicity assay. Previous work from our laboratory and others has demonstrated that tumor cells treated with zoledronate are aggressively killed by γδ T cells (10, 77). Our data demonstrate that treatment of HDAC inhibitors retard the ability of γδ T cells to kill zoledronate-treated tumor targets. Further, we proved that this inhibition of cytotoxic potential of γδ T cells was due to decrease in the expression of perforin and granzyme-B in these cells.

The activation of T cells initiated through T cell receptor is regulated by balance between co-stimulatory and inhibitory signals (immune checkpoints). Imbalance between these signals lead to different pathological conditions like tumor. Majority of the tumors use these immune checkpoints such as PD-1 or its ligand PD-L1 to escape from the immune surveillance. Immune check point inhibitors have revolutionized the field of tumor immunotherapy (34). Besides surgery, radiation, and chemotherapy, immune check point inhibitors have surfaced as an important immunotherapeutic approach for cancer treatment. Due to their promising antitumor effects in experimental animal models, preclinical studies and successful clinical trials, immune check point inhibitors have been now approved by the U.S Food and Drug Administration (FDA) for treatment of different malignancies. PD-1/PD-L1 blocking strategy has led to tumor regression in patients with melanoma, renal cell carcinoma, non-small cell lung cancer, and bladder cancer (78–82).

Recent reports have shown that tumors associated with PD-1 expressing NK cells show poor survival (83). PD-1/PD-L1 signaling axis along with NKG2D signaling axis determine effector response of NK cells. Blockade of PD1/PD-L1 signaling cascade in NK cells along with other antitumor drugs have shown promising responses in cancer patients (84). This study supports our observation that HDAC inhibitors modulate the effector functions of human γδ T cells against tumors *via* PD1/PD-L1 signaling axis. We observed that γδ T cells show increased expression of immune check points PD1 and PD-L1 upon HDAC inhibitor treatment.

A report by Garcia-Diaz et al. have shown that induction of PD-L1 and PD-L2 on tumor cells is regulated *via* IFN-γ (85). In the present study, we have demonstrated that HDAC inhibitors decrease the expression of IFN-γ and TNF-α in antigen-activated γδ T cells. It has been demonstrated that Tbet transcription factor binds to PD-1 promoter and mediates the suppression of PD-1 expression (86). In the present study, we have shown that upon HDAC inhibitor treatment of γδ T cells, Tbet protein and mRNA is decreased significantly indicating that less Tbet may be available to bind PD-1 promoter to suppress PD-1 expression. This mechanism may explain the IFN-γ independent mechanism of PD-1 expression on γδ T cells.

Activated γδ T cells are known to express PD-1, which was investigated by Iwaski et al., on expanded γδ T cells population. They found that γδ T cells express PD-1 rapidly from day 3 of induction and PD-1^+^ γδ T cells exhibit attenuated effector functions and decreased cytotoxicity against PD-L1 expressing tumors. However, they observed that zoledronate treatment to tumor cells, which induces IPP release along with PD-L1 blockade, rescued the γδ T cell cytotoxicity (35). While our study also confirms that blocking of PD-1 in γδ T cells increases the antitumor cytotoxic potential, our study reports on the effect of HDAC inhibitors on the freshly isolated γδ T cells activated with antigen for 72 h, whereas Iwaski group used γδ T cells already in activation state for their experimental purposes. Another interesting study by Castella et al. explores the multifunctional role of zoledronate in augmenting γδ T cells responses against multiple myeloma. In this study, zoledronate-treated autologous DCs were found to efficiently activate γδ T cells and enhance their cytotoxic functions against myeloma cells. Additionally, zoledronate was also shown to promote antitumor immunity *via* suppression of regulatory T cell function, downregulation of PD-L1 expression on DCs, and increased proliferation of tumor antigen-specific CD8 T cells. Although, their study has effectively demonstrated role of zoledronate in enhancing antitumor responses γδ T cells, it is specific only to multiple myeloma and uses zoledronate expanded γδ T cells from patient PBMNCs (87). Converse to our observation, they found that DC-activated γδ T cells did not express PD-1, this might be due to the immune modulation by zoledronate, which needs further exploration.

We observed that blockade of PD1/PD-L1 signaling partially restores the antitumor cytotoxic function of γδ T cells in the presence of HDAC inhibitors, which reflected in increased expression of effector molecules granzyme B and Lamp-1. Wei et al. have demonstrated that PD-1 ligation dramatically shifts the dose–response curve, making CD8+ Tcells much less sensitive to TCR generated signals (88). Although, this was shown in CD8+ αβ T cells, it may also apply to γδ T cells. Thus, PD-1 ligation affects TCR signaling and thereby reduces the cytotoxic function of γδ T cells. The role of other activating receptors such as NKG2D interacting with MICA/B and inhibitory receptors KIR2DL2/3 (CD158b) cannot be ignored and it explains the incomplete restoration of cytotoxic effector function γδ T cells upon PD-1 blocking.

Our results implicate that HDAC inhibitors along with the immune checkpoint modulating antibodies could be developed as combination immunotherapy to treat different malignancies. Thus, in future, this strategy may be applied for overcoming the limitations of HDAC inhibitor-based cancer therapies. The underlying mechanistic link of PD-1/PD-L1 may be targeted in developing more efficacious combination γδ T cell-based therapies in the future.

## Ethics Statement

The study was approved by the Institutional Ethics Committee of ACTREC-TMC. All subjects gave written informed consent in accordance with the Declaration of Institutional Ethics Committee, ACTREC-TMC.

## Author Contributions

SC supervised, contributed conceptionally, and helped to write the manuscript. SB conducted experiments and wrote the manuscript. DV helped in conducting experiments. All authors contributed in final approval of manuscript.

## Conflict of Interest Statement

The authors declare that the research was conducted in the absence of any commercial or financial relationships that could be construed as a potential conflict of interest.

## Supplementary Material

Figure S1 Histone deacetylases (HDAC) inhibitors and viability of γδ T cells. HDAC inhibitors affect viability of γδ T cells only beyond specific concentrations. γδ T cells stimulated with HDMAPP in the presence of rIL-2 were treated with HDAC inhibitors sodium valproate (4, 3, 2, 1, and 0.5 mM), Trichostatin-A (250, 150, 100, 50, and 25 nM), and suberoylanilidehydroxamic acid (4, 3, 2, 1, 0.5 µM) for 72 h. The viability of γδ T cells was assessed by MTT assay. The results indicated are mean ± SE percent viability of γδ T cells and are representative of three experiments.

Figure S2 The effect of histone deacetylases (HDAC) inhibitors on γδ T cells viability. **(A)** Purity of sorted γδ T cells. γδ T cells were positively sorted from peripheral blood mononuclear cells and were positive for γδ T-cell receptor (TCR) (90.8%), CD56 (53.2%), and negative for αβTCR, CD14, CD19. **(B)** Effect of HDAC inhibitor treatment on viability of γδ T cells. γδ T cells were activated with HDMAPP and rIL-2. HDAC inhibitors sodium valproate (2, 1, and 0.5 mM), Trichostatin-A (100, 50, and 25 nM), and suberoylanilidehydroxamic acid (1, 0.5, and 0.25 µM) were added to the culture and apoptosis was measured after 72 h with Annexin V and 7-AAD staining. Data shown are representative of three independent experiments. **(C)** The graphs show consolidated γδ T cell viability post HDAC inhibitor treatment (*n* = 3). Data represent mean ± SE.

Figure S3 Histone deacetylases (HDAC) inhibitors impede proliferation of γδ T cells in a dose-dependent manner and leads to cell cycle arrest in G0–G1 phase. **(A)** The proliferative response of γδ T cells was assessed by thymidine incorporation assay. Sorted γδ T cells were stimulated with phosphoantigen HDMAPP, with or without treatment with HDAC inhibitors sodium valproate, Trichostatin-A, and suberoylanilidehydroxamic acid at different concentrations for 72 h. The graphs illustrate the cumulative mean cpm and are representative of three independent experiments where **p* < 0.05, ***p* < 0.005, ****p* < 0.0005. **(B)** Cell cycle analysis of γδ T cells upon HDAC inhibitor treatment. Freshly isolated γδ T cells were activated with HDMAPP with or without HDAC inhibitors for 72 h, and cell cycle progression was analyzed with propidium iodide (PI) staining using FACS calibur. The histograms are representative of three independent experiments. **(C)** The graphs indicate cumulative mean percentage of PI-positive cells in each phase of cell cycle. The graphs are representative of three independent experiments where **p* < 0.05, ***p* < 0.005, ****p* < 0.0005.

Figure S4 Histone deacetylases (HDAC) inhibitors differentially affects the expression of activating and inhibitory receptors in γδ T cells. The expression of **(A)** NKG2D, **(B)** KIR2D2L2/3, and **(C)** CD16 by γδ T cells in the presence and absence of HDAC inhibitors after 72 h was analyzed by flow cytometry. The histograms are representative of three individual experiments. The values on right side of histograms indicate median fluorescence intensity. HDAC inhibitors reduce NFκB expression in γδ T cells. Protein expression of NFκB by γδ T cells upon treatment with **(C)** sodium valproate, **(D)** Trichostatin-A, and **(E)** suberoylanilidehydroxamic acid as detected by western blotting. Cell lysates of γδ T cells, stimulated with HDMAPP after treatment with HDAC inhibitors at different concentrations for 72 h were probed with NFκB antibody, β-actin was used as loading control. The blots shown are representative of three experiments.

Figure S5 Histone deacetylases (HDAC) inhibitors decrease the acetylation on promoters of perforin and granzyme B in γδ T cells. **(A)** γδ T cells treated with HDAC inhibitors sodium valproate (VPA), Trichostatin-A (TSA), and suberoylanilidehydroxamic acid (SAHA) show increased acetylation of H3 and H4. Expression of acetyl Histone 3 and acetyl histone 4 in γδ T cells was detected by western blotting and total H3 and total H4 were used as loading control. The blots shown are representative of three experiments. Chromatin immunoprecipitation qPCR was used to analyze the histone H3 acetylation **(B)** and histone H4 acetylation **(C)** on perforin region I (−103 to +27 bp), perforin region II (−309 to −130 bp); granzyme B region I (−130 to +11 bp), and granzyme B region II (−263 to −77 bp) from the transcription start site (TSS) of perforin and granzyme B in γδ T cells treated with or without HDAC inhibitors VPA (2 mM), TSA (100 nM), and SAHA (1 µM) for 72 h. Enrichment of promoter regions of Perforin and Granzyme B was quantified by qPCR and expressed as percentage of input DNA. The images above the graphs are schematic representation of Perforin and granzyme B promoter regions including the TSS. All results indicated are mean + SEM where **p* < 0.05, ***p* < 0.005, ****p* < 0.0005.
