# Supplementary figures and images for: Checkpoint Blockade Rescues the Repressive Effect of Histone Deacetylases Inhibitors on γδ T Cell Function
Source: Front Immunol. 2018 Jul 19;9:1615. doi: 10.3389/fimmu.2018.01615 (PMC6060239; doi:10.3389/fimmu.2018.01615)

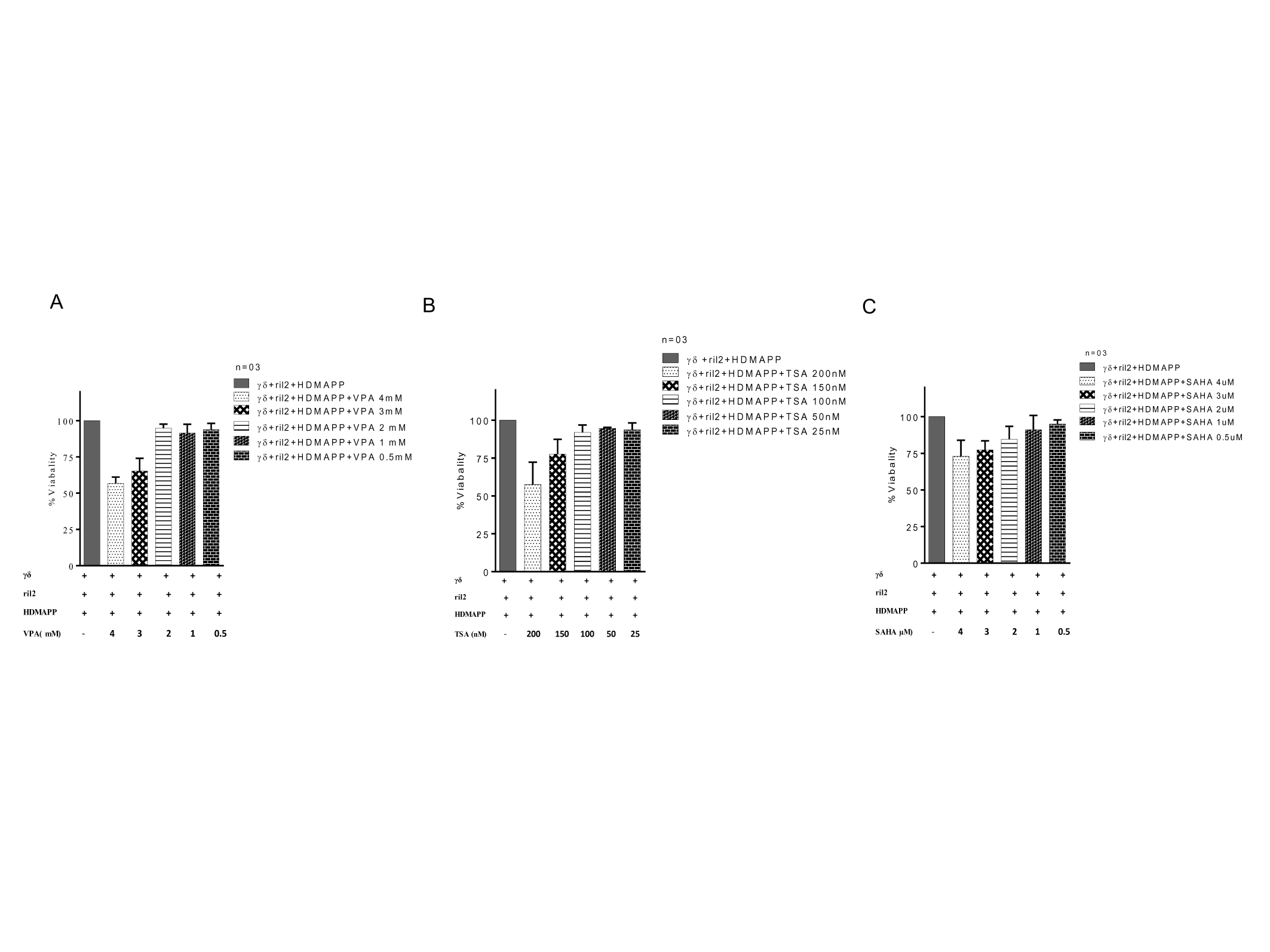

Supplement: Figure S1 — Histone deacetylases (HDAC) inhibitors and viability of γδ T cells. HDAC inhibitors affect viability of γδ T cells only beyond specific concentrations. γδ T cells stimulated with HDMAPP in the presence of rIL-2 were treated with HDAC inhibitors sodium valproate (4, 3, 2, 1, and 0.5 mM), Trichostatin-A (250, 150, 100, 50, and 25 nM), and suberoylanilidehydroxamic acid (4, 3, 2, 1, 0.5 µM) for 72 h. The viability of γδ T cells was assessed by MTT assay. The results indicated are mean ± SE percent viability of γδ T cells and are representative of three experiments. [file image_1.tif]

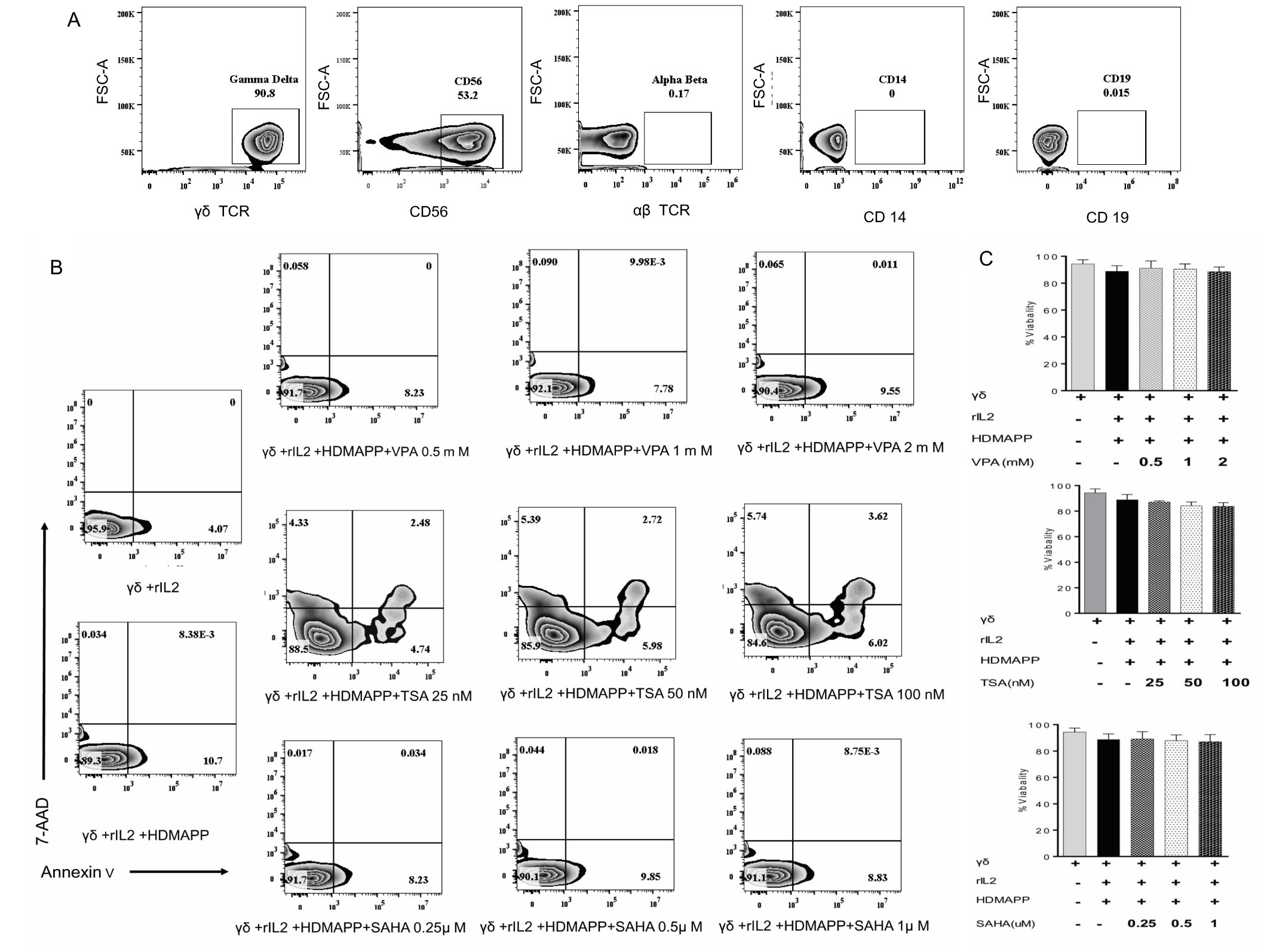

Supplement: Figure S2 — The effect of histone deacetylases (HDAC) inhibitors on γδ T cells viability. (A) Purity of sorted γδ T cells. γδ T cells were positively sorted from peripheral blood mononuclear cells and were positive for γδ T-cell receptor (TCR) (90.8%), CD56 (53.2%), and negative for αβTCR, CD14, CD19. (B) Effect of HDAC inhibitor treatment on viability of γδ T cells. γδ T cells were activated with HDMAPP and rIL-2. HDAC inhibitors sodium valproate (2, 1, and 0.5 mM), Trichostatin-A (100, 50, and 25 nM), and suberoylanilidehydroxamic acid (1, 0.5, and 0.25 µM) were added to the culture and apoptosis was measured after 72 h with Annexin V and 7-AAD staining. Data shown are representative of three independent experiments. (C) The graphs show consolidated γδ T cell viability post HDAC inhibitor treatment (n = 3). Data represent mean ± SE. [file image_2.tif]

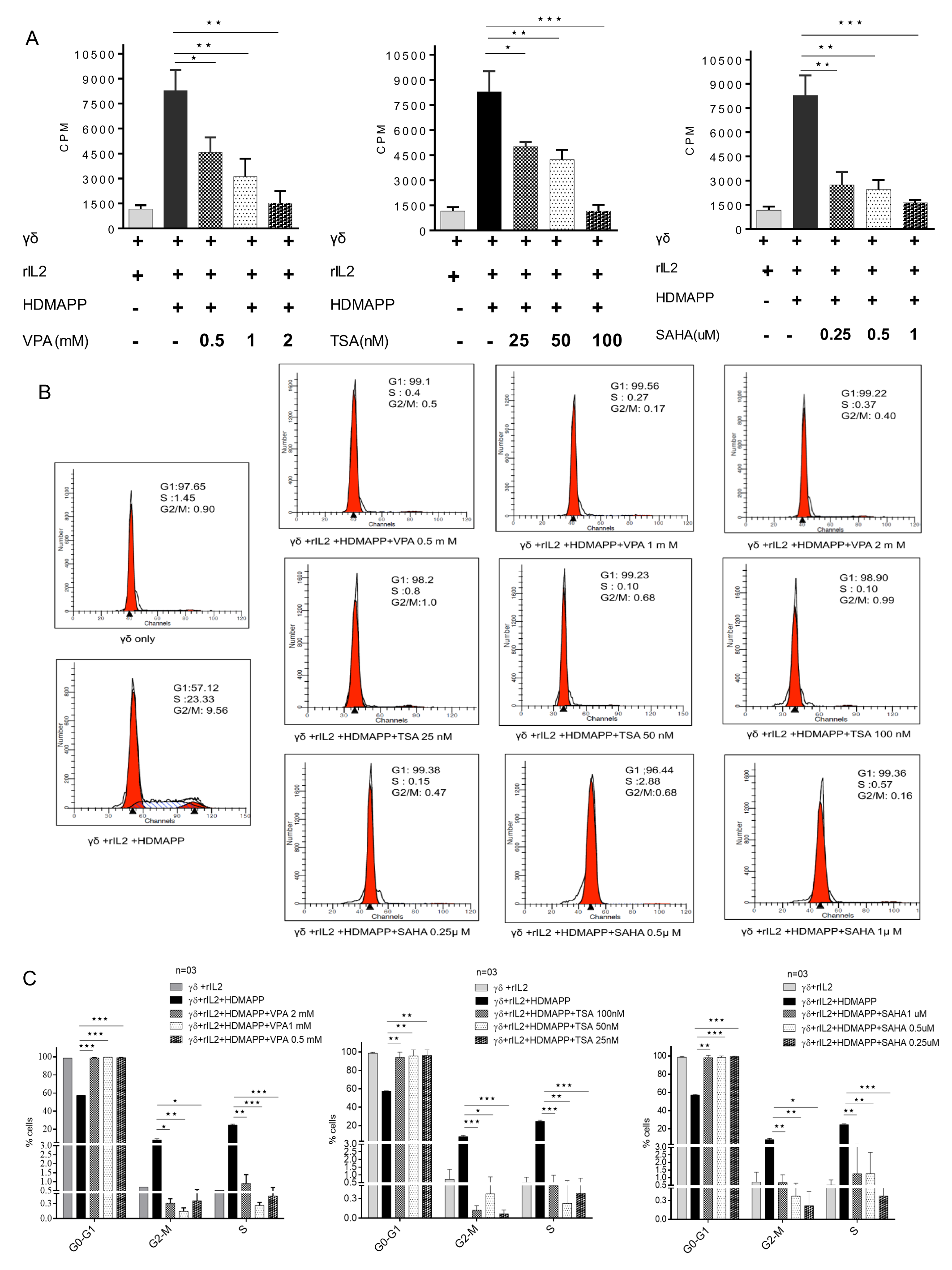

Supplement: Figure S3 — Histone deacetylases (HDAC) inhibitors impede proliferation of γδ T cells in a dose-dependent manner and leads to cell cycle arrest in G0–G1 phase. (A) The proliferative response of γδ T cells was assessed by thymidine incorporation assay. Sorted γδ T cells were stimulated with phosphoantigen HDMAPP, with or without treatment with HDAC inhibitors sodium valproate, Trichostatin-A, and suberoylanilidehydroxamic acid at different concentrations for 72 h. The graphs illustrate the cumulative mean cpm and are representative of three independent experiments where *p < 0.05, **p < 0.005, ***p < 0.0005. (B) Cell cycle analysis of γδ T cells upon HDAC inhibitor treatment. Freshly isolated γδ T cells were activated with HDMAPP with or without HDAC inhibitors for 72 h, and cell cycle progression was analyzed with propidium iodide (PI) staining using FACS calibur. The histograms are representative of three independent experiments. (C) The graphs indicate cumulative mean percentage of PI-positive cells in each phase of cell cycle. The graphs are representative of three independent experiments where *p < 0.05, **p < 0.005, ***p < 0.0005. [file image_3.tif]

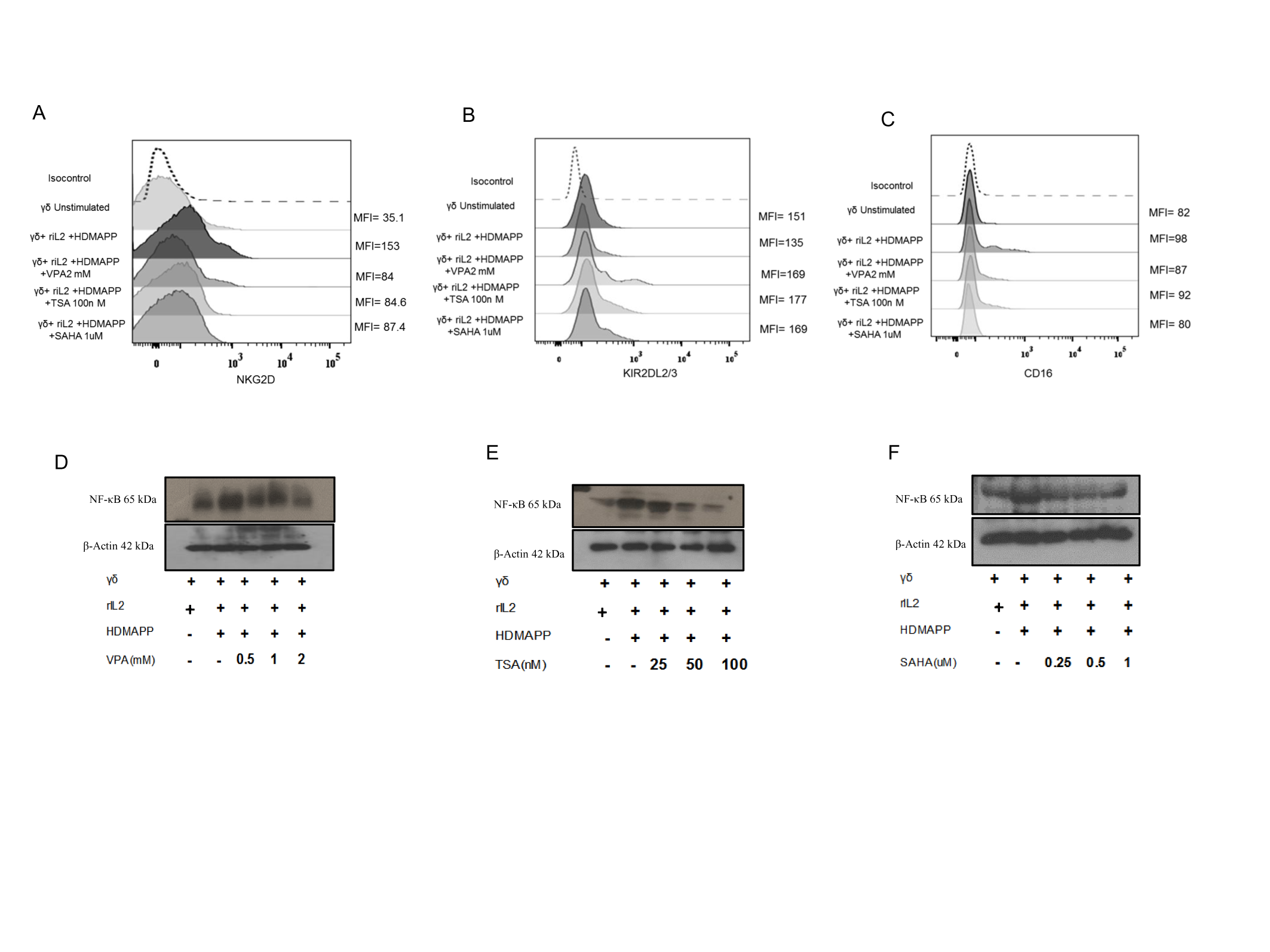

Supplement: Figure S4 — Histone deacetylases (HDAC) inhibitors differentially affects the expression of activating and inhibitory receptors in γδ T cells. The expression of (A) NKG2D, (B) KIR2D2L2/3, and (C) CD16 by γδ T cells in the presence and absence of HDAC inhibitors after 72 h was analyzed by flow cytometry. The histograms are representative of three individual experiments. The values on right side of histograms indicate median fluorescence intensity. HDAC inhibitors reduce NFκB expression in γδ T cells. Protein expression of NFκB by γδ T cells upon treatment with (C) sodium valproate, (D) Trichostatin-A, and (E) suberoylanilidehydroxamic acid as detected by western blotting. Cell lysates of γδ T cells, stimulated with HDMAPP after treatment with HDAC inhibitors at different concentrations for 72 h were probed with NFκB antibody, β-actin was used as loading control. The blots shown are representative of three experiments. [file image_4.tif]

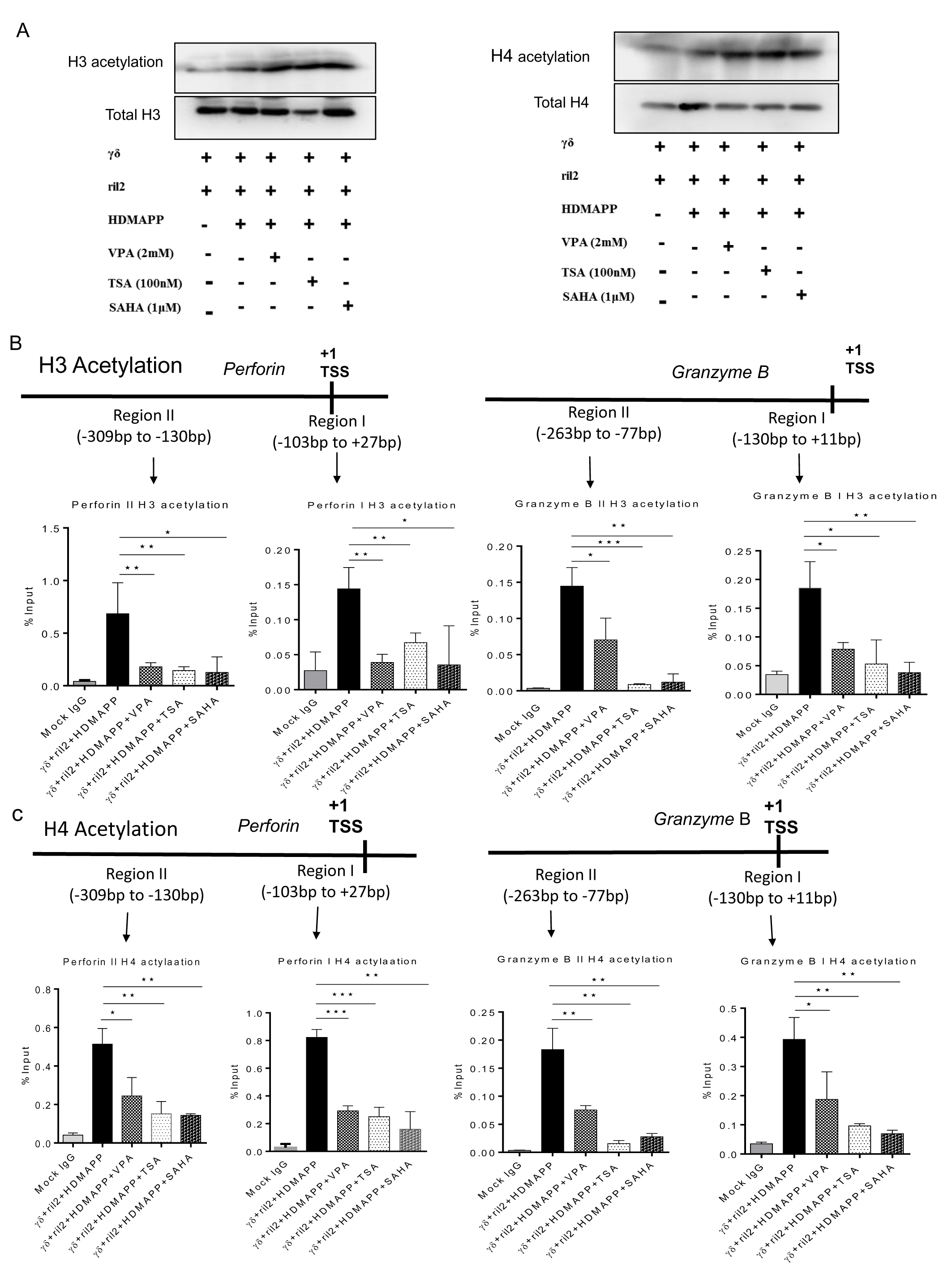

Supplement: Figure S5 — Histone deacetylases (HDAC) inhibitors decrease the acetylation on promoters of perforin and granzyme B in γδ T cells. (A) γδ T cells treated with HDAC inhibitors sodium valproate (VPA), Trichostatin-A (TSA), and suberoylanilidehydroxamic acid (SAHA) show increased acetylation of H3 and H4. Expression of acetyl Histone 3 and acetyl histone 4 in γδ T cells was detected by western blotting and total H3 and total H4 were used as loading control. The blots shown are representative of three experiments. Chromatin immunoprecipitation qPCR was used to analyze the histone H3 acetylation (B) and histone H4 acetylation (C) on perforin region I (−103 to +27 bp), perforin region II (−309 to −130 bp); granzyme B region I (−130 to +11 bp), and granzyme B region II (−263 to −77 bp) from the transcription start site (TSS) of perforin and granzyme B in γδ T cells treated with or without HDAC inhibitors VPA (2 mM), TSA (100 nM), and SAHA (1 µM) for 72 h. Enrichment of promoter regions of Perforin and Granzyme B was quantified by qPCR and expressed as percentage of input DNA. The images above the graphs are schematic representation of Perforin and granzyme B promoter regions including the TSS. All results indicated are mean + SEM where *p < 0.05, **p < 0.005, ***p < 0.0005. [file image_5.tif]
